# Dynamic single-cell RNA-seq analysis reveals distinct tumor program associated with microenvironmental remodeling and drug sensitivity in multiple myeloma

**DOI:** 10.1186/s13578-023-00971-2

**Published:** 2023-01-30

**Authors:** Mengping Chen, Yike Wan, Xin Li, Jing Xiang, Xiaotong Chen, Jinxing Jiang, Xiaofeng Han, Lu Zhong, Fei Xiao, Jia Liu, Honghui Huang, Hua Li, Junling Liu, Jian Hou

**Affiliations:** 1 Department of Hematology, Ren Ji Hospital, Shanghai Jiao Tong University School of Medicine, Shanghai, 200127 China; 2 Bio-ID Center, Shanghai Jiao Tong University School of Biomedical Engineering, Shanghai, 200240 China; 3 Department of Biochemistry and Molecular Cell Biology, Shanghai Jiao Tong University School of Medicine, Shanghai, 200025 China

**Keywords:** Multiple myeloma, Single-cell RNA sequencing, Transcriptional profiling, Drug sensitivity, Tumor microenvironment

## Abstract

**Background:**

Multiple myeloma (MM) is a hematological malignancy characterized by clonal proliferation of malignant plasma cells. Despite extensive research, molecular mechanisms in MM that drive drug sensitivity and clinic outcome remain elusive.

**Results:**

Single-cell RNA sequencing was applied to study tumor heterogeneity and molecular dynamics in 10 MM individuals before and after 2 cycles of bortezomib–cyclophosphamide–dexamethasone (VCD) treatment, with 3 healthy volunteers as controls. We identified that unfolded protein response and metabolic-related program were decreased, whereas stress-associated and immune reactive programs were increased after 2 cycles of VCD treatment. Interestingly, low expression of the immune reactive program by tumor cells was associated with unfavorable drug response and poor survival in MM, which probably due to downregulation of MHC class I mediated antigen presentation and immune surveillance, and upregulation of markers related to immune escape. Furthermore, combined with immune cells profiling, we uncovered a link between tumor intrinsic immune reactive program and immunosuppressive phenotype in microenvironment, evidenced by exhausted states and expression of checkpoint molecules and suppressive genes in T cells, NK cells and monocytes. Notably, expression of YBX1 was associated with downregulation of immune activation signaling in myeloma and reduced immune cells infiltration, thereby contributed to poor prognosis.

**Conclusions:**

We dissected the tumor and immune reprogramming in MM during targeted therapy at the single-cell resolution, and identified a tumor program that integrated tumoral signaling and changes in immune microenvironment, which provided insights into understanding drug sensitivity in MM.

**Supplementary Information:**

The online version contains supplementary material available at 10.1186/s13578-023-00971-2.

## Background

Multiple myeloma is a hematological malignancy with accumulation of clonal plasma cells (PCs) in the bone marrow (BM) [1]. The present treatment using proteasome inhibitors (PIs), immunomodulatory drugs (IMiDs) and monoclonal antibodies elicits deep remission and prolonged survival in newly diagnosed MM (NDMM) [2]. However, MM remains an incurable disease, with almost all patients relapse eventually [3]. A unique feature of this malignancy is the clonal heterogeneity including both primary and secondary cytogenetic abnormalities as well as specific molecular alterations, which have been demonstrated to influence therapeutic outcomes [4–7]. Previous attempts to unravel the tumor complexity at single-cell resolution have revealed significant inter-patient heterogeneity across disease spectrum from precursor asymptomatic disease stages to active MM [8] and even the relapsed/refractory multiple myeloma (RRMM) stage [9, 10]. In addition to these inter-tumor heterogeneities, MM also displays enormous intra-tumor heterogeneity (ITH) comprised of a mixture of clones [4, 11, 12] and diverse transcriptional programs [10, 13], which pose both challenges and opportunities for myeloma therapy.

PIs (e.g., bortezomib, carfilzomib) are impressively effective for myeloma by targeting ubiquitin–proteasome system and are routinely used in combination with other anti-myeloma agents [14–16]. However, not all patients respond equally well to treatment with drugs, including PIs, and patients often acquire therapeutic resistance over the course of treatment, which remains a crucial obstacle to improve therapeutic effect in MM [17, 18]. Clonal selection and evolution play roles in drug response and disease progression in MM [7, 19, 20]. Chemotherapy affects transcriptional programs and clone evolution of myeloma cells, which provides an opportunity to systematically decipher the most relevant treatment-induced cellular responses and may help to define novel effectively targeted therapeutics. In addition, prior studies have confirmed compositional and expression changes of immune and stromal components in BM microenvironment associated with anti-myeloma responses and therapeutic outcomes [9, 21, 22]. Collectively, these intrinsic and extrinsic factors highlight the need to understand the molecular mechanisms underlying the drug response, disease heterogeneity and progression of MM.

Single-cell RNA-seq (scRNA-seq) methodologies are extending our ability to dissect myeloma cell heterogeneity and define dynamic changes of their microenvironment in a high-resolution way [8, 9, 13, 23]. However, the tumor and microenvironment determinants of response to anti-myeloma agents remain incompletely understood. In this work, we performed scRNA-seq to analyze tumor heterogeneity and molecular dynamics of 10 individuals with MM before and after VCD treatment, as well as 3 healthy volunteers. Using this unique paired resource, we analyzed cellular heterogeneity and transcriptional reprogramming by quantifying variations in oncogenic signaling pathways, as well as microenvironmental states during the therapy, thereby providing potential mechanisms in MM pathogenesis and treatment response.

## Results

### Single-cell expression atlas and cell typing in MM patients and healthy donors

**Fig. 1:**
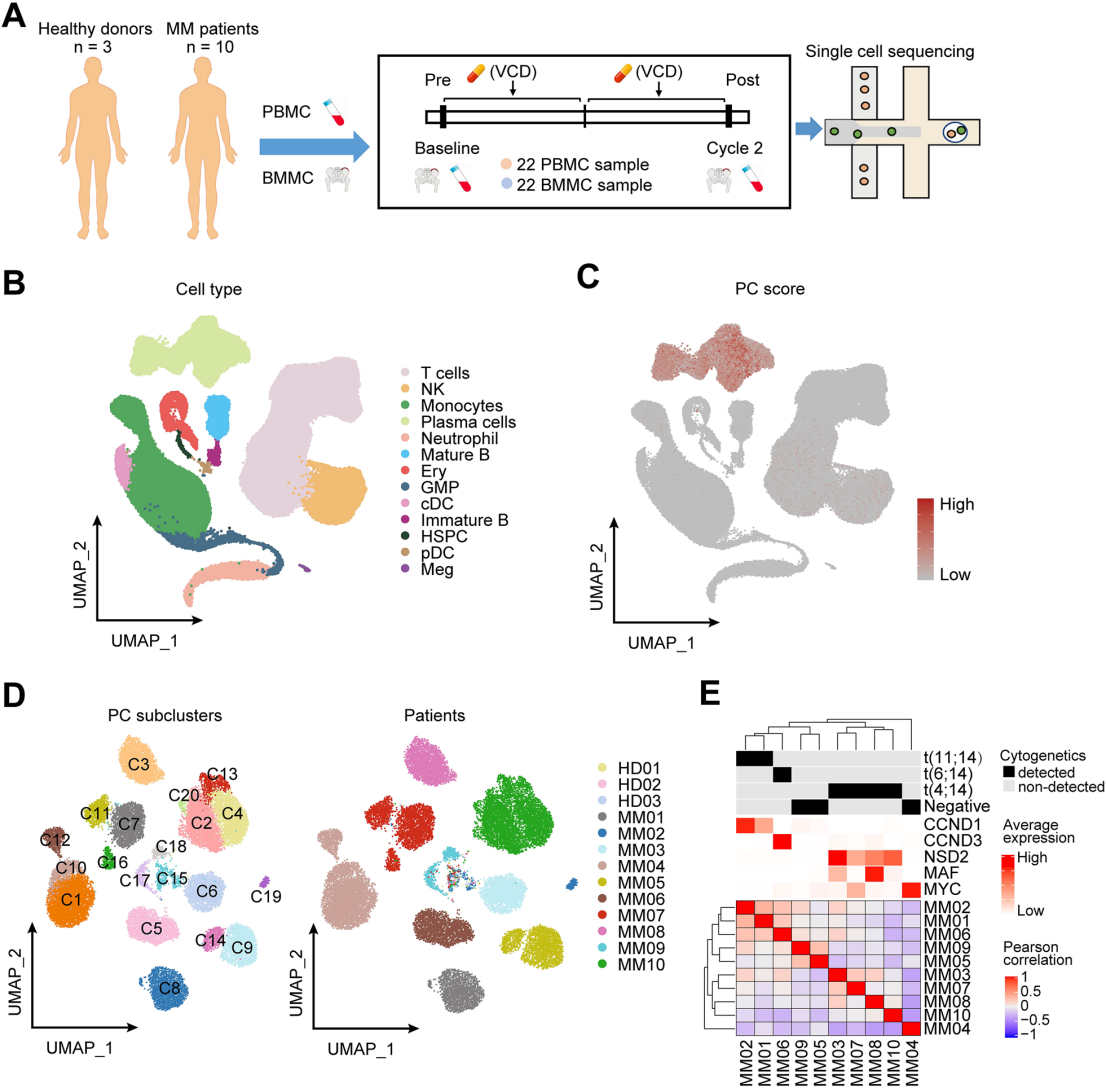
Single cell assessment revealed tumor heterogeneity in myeloma. **A** Schematic representation of the experimental strategy and sampling time-points of the study. **B** UMAP plot of single cells profiled in the presenting work colored by major cell types after fastMNN integration. **C** UMAP plot of single cells colored by plasma cell (PC) score according to expression of the plasma cell markers (SDC1/CD138, TNFRSF17 and SLAMF7). **D** UMAP plots showing the re-clustering of plasma cell (PC) colored by subclusters (left) and patients (right). **E** Pearson correlation matrix of averaged gene expression levels per patient. Top, cytogenetic information; bottom, averaged gene expression levels of five MM driver genes

To define transcriptional states in MM at single-cell resolution, we used 10× Genomics to perform scRNA-seq of mononuclear cells from the BM and peripheral blood (PB) of 10 NDMM patients and 3 healthy volunteers (Fig. 1A). MM patients were treated with VCD regimen, and paired samples were collected at baseline and 2 cycles after treatment. In total, 10 pre- and 9 post-treatment BM and corresponding PB samples from MM patients, as well as 3 BM and 3 PB from healthy volunteers were collected and processed for scRNA-seq analysis (Additional file 1: Table S1). By comparing different batch-effect correction methods, we achieved a better integration result by fastMNN [24] in terms of the ability to integrate batches while maintaining cell type separation (Fig. 1B, Additional file 1: Figs. S1A, B and S2A–C). After quality filtering, we obtained the single-cell transcriptome data for 241,440 high-quality mononuclear cells, and identified 13 major cell types including T cells, NK cells, plasma cells, myeloid cells, B cells and precursor cells based on well-established marker genes (Fig. 1B, Additional file 1: Fig. S2A–C). Two-dimensional embedding using UMAP showed a clear separation of immune cells from plasma/myeloma cells with the high PC scores defined by expression of the plasma cell markers (Fig. 1C). Overall, we profiled 25,231 plasma cells and 216,209 immune cells for an integrated analysis of both tumor and immune-cell heterogeneities in MM. In contrast to PB, several precursor cells were enriched in BM, including HSPC (hematopoietic stem and progenitor cell), GMP (granulocyte–monocyte progenitor), Ery (erythroid progenitors) and immature B cells. However, other major immune cells including T/NK cells, monocytes, dendritic cells (DC) and mature B cells were enriched in PB (Additional file 1: Fig. S2C). When analyzed cell type abundances during treatment, we observed that besides plasma cells, B cells proportion was also reduced in patients treated with VCD (Additional file 1: Fig. S2C), which was also found in MM patients after IMiD-based treatments [9, 25], reflecting their common drug vulnerabilities likely due to the close lineage.

As MM typically presents with BM infiltration of clonal plasma cells, myeloma cells in each patient enriched in BM, with few cells derived from PB (Additional file 1: Fig. S3A, B). Comparing the circulating myeloma cells with the BM myeloma cells for each subject, we observed that in most cases, the circulating tumor cell signatures highly resembled the BM transcriptional profiles, except for one patient (MM04) with the highest abundance of PB tumor cells (Additional file 1: Fig. S3C). Compared with BM, circulating myeloma cells from this patient exhibited high expression of genes that correlated with disease aggressiveness and cell migration (Additional file 1: Fig. S3D), which may explain their presences in circulation.

### Identification of inter- and intral-tumor heterogeneity in MM

To better understand the transcriptional features in normal plasma cells (nPCs) and myeloma cells, we re-clustering PCs and characterized them into 20 different clusters (Fig. 1D). The clustering results revealed a strong transcriptional heterogeneity between MM patients, while nPCs from healthy volunteers and small proportion of cells in MM clustered together in cluster 15, suggesting there were several nPCs in MM samples (Fig. 1D). We then distinguished myeloma cells from nPCs by inferCNV tool [26]. We estimated copy number alterations (CNAs), using nPCs from healthy donors as reference and profiled CNAs of plasma/myeloma cells from each MM patient, and we were able to robustly distinguish myeloma cells from nPCs (Additional file 1: Fig. S4A–C). Myeloma cells highly expressed well-known driver genes, including CCND1, NDS2/MMSET, CCND3 and pathway analysis showed prominent upregulations of multiple biological processes and pathways in myeloma cells compared with nPCs (Additional file 1: Fig. S5A, B). Then, the gene expression-based hierarchical clustering classified myeloma cells into transcriptional subtypes consistent with their cytogenetics, which was also evident by their driver genes and other essential genes expression pattern in each immunoglobulin heavy chain (*IGH*) translocation group (Fig. 1E, Additional file 1: Fig. S5C, D). These results suggested inter-myeloma heterogeneity could be explained by well-known oncogenic drivers and distinct transcriptional signatures.

To further investigate intral-tumor heterogeneity (ITH), we performed clustering analysis on PCs for each MM individually and combined their CNA clones to inspect overall ITH. We observed that the number of transcriptional clusters per patient increased with the number of cells analyzed, however CNA clone number didn’t show significant correlation with cluster number (Additional file 1: Fig. S6A), which was consistent with previous scRNA-seq study [9]. We next determined ITH score of tumor cells in each MM patient based on their gene expression profiles through DEPTH package [27], and found that ITH score was apparently different in each patient (Additional file 1: Fig. S6B). However, average ITH score showed no significant correlations either with the cluster number or CNA clone number (Additional file 1: Fig. S6B). We next tested association of ITH score with clinical characteristics of MM patients from CoMMpass dataset with a larger sample size. Results showed that ITH score was significantly higher in relapsed MM compared with NDMM (Additional file 1: Fig. S6C), and survival analyses indicated that higher ITH score was associated with worse overall survival (OS) and progression-free survival (PFS) (Additional file 1: Fig. S6D, E). Together, these results showed that ITH was prevalent in myeloma which can be predictive for clinic outcome.

### Four malignant cell programs dysregulated after treatment

**Fig. 2:**
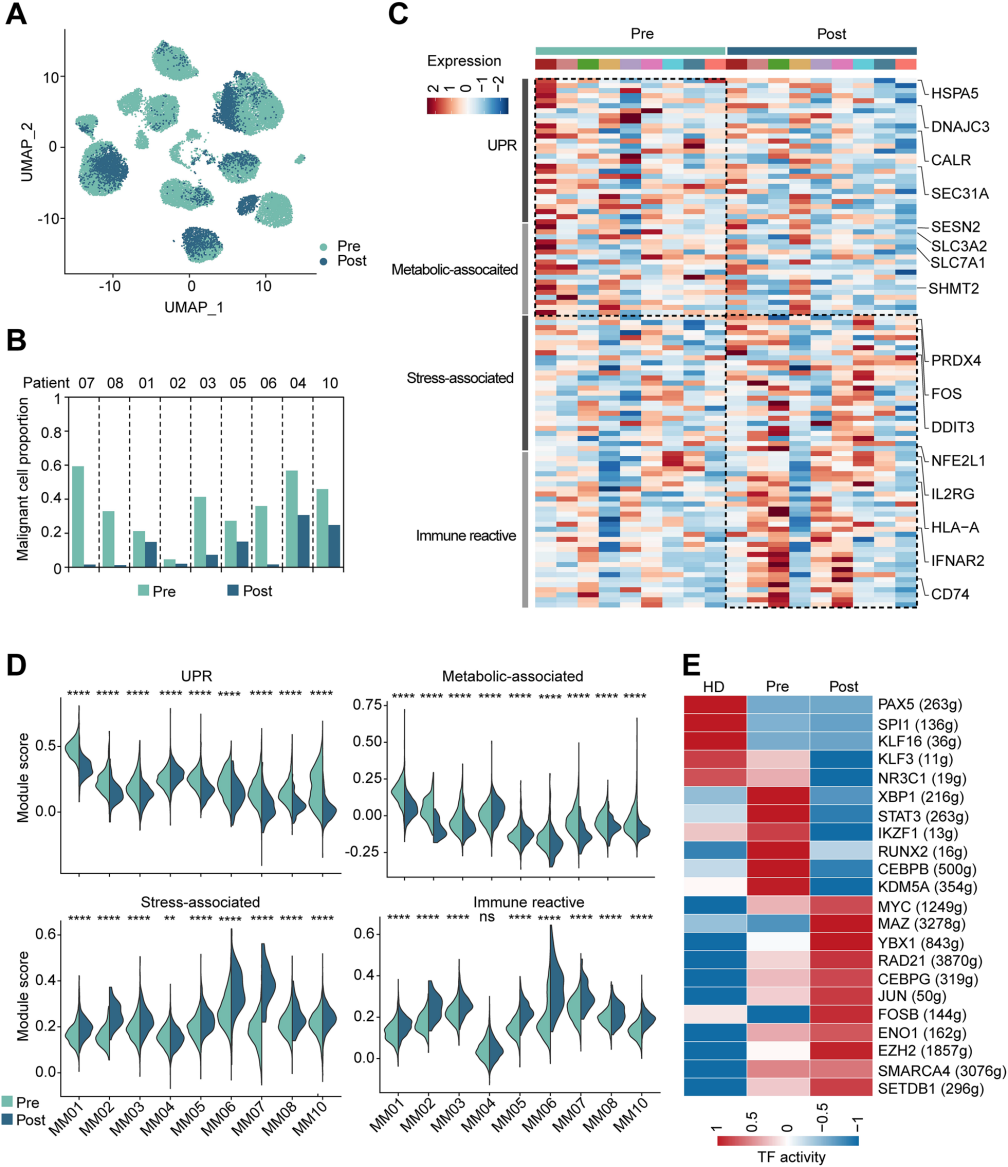
Treatment induced transcriptional changes in tumor cells. **A** UMAP embedding of myeloma cells colored by treatment time points (Pre and Post). **B** Bar plot of myeloma cell fraction in 9 matched pre and post samples. **C** Heat map of average expression of representative genes and corresponding pathways in pre and post-treatment samples. **D** Scores of UPR, metabolic-associated, stress-associated and immune reactive programs in each MM patient with matched pre and post samples. **E** Heat map of the area under the curve (AUC) scores of TF motifs estimated per cell by SCENIC. Shown are representative differentially activated motifs in nPCs (HD), myeloma cell pre and post-treated, respectively. Statistical analysis in **D** was performed by Wilcoxon test. HD, n = 3; pre, n = 9, post = 9; ns, not significant; **p < 0.01, ****p < 0.0001

Drug-induced changes may have the potential to reveal important insights into the molecular mechanisms in action of chemotherapeutics. Therefore, we set out to investigate the global gene expression profiling of myeloma cells before and after VCD treatment. After 2 cycles of treatment, each patient exhibited different extent of reductions in tumor cells abundance due to the effectiveness of VCD induction therapy (Fig. 2A, B, Additional file 1: Fig. S7A), and most of patients achieved very good partial response (VGPR) or partial response (PR) (Additional file 1: Table S1) defined by International Myeloma Working Group (IMWG) criteria [28]. Interestingly, ITH score also showed a significant reduction after treatment, probably due to the elimination of tumor cells (Additional file 1: Fig. S7B). When comparing the driver genes such as CCND1, CCND3, NDS2/MMSET in each patient, we found that among the four MM with t(4;14) translocation, two patients (MM07, MM08) with VGPR showed dramatic reductions in FGFR3 expression after treatment, however other two patients with PR or stable disease (SD) (MM03 and MM10 respectively) showed this decrease in a less extent (Additional file 1: Fig. S7C). A remarkable decrease in CCND1 expression was also found in one of the MM patients with t(11;14) (MM02) who achieved PR post treatment, which may suggest a potential link between reduction of driver genes expression and drug response.

Based on the great inter-tumor heterogeneity, to profile dynamic transcriptional changes, we determined differentially expressed genes (DEGs) between per- and post-treated tumor cells within the same subject respectively and detected 163 to 520 DEGs in each patient. We then performed functional enrichment analysis and identified four cellular programs shared across MM patients affected by chemotherapy, including unfolded protein response (UPR), metabolic-associated, stress-associated and immune reactive programs (Fig. 2C). UPR program was characterized by key genes regulating protein folding and endoplasmic reticulum (ER) stress, and metabolic-associated program was featured by high expression of metabolic genes involved in glycolysis and amino acids metabolism (Fig. 2C and Additional file 1: Table S2). Expressions of these two programs were consistently reduced in post-treated samples (Fig. 2D), implied that chemotherapy either killed most of the tumor cells with high UPR and metabolic state or induced inhibition in these cell states. Stress-associated signature consisted of stress-responsive genes (e.g., FOS JUN), pro-survival (e.g., GADD45A, MCL1 and BCL2) and oxidative stress genes (e.g., PRDX4 and GCSH) (Fig. 2C and Additional file 1: Table S2). Increase in stress program after treatment (Fig. 2D) suggested that chemotherapy elicited stress response in tumor cells and may in turn assist myeloma in anti-apoptosis and recovery from drug targeting, supporting the finding that bortezomib triggers oxidative stress response [29–31] and high cellular antioxidant capacity thereby contributing to drug resistance [31, 32]. Additional immune reactive program contained various genes associated with immune response and activation pathways such as antigen presentation (e.g., HLA-A, HLA-B, HLA-C, CD74, CTSS), interferon (IFN) signaling (e.g., IRF1, IFIH1, IFITM1, IFNAR1), chemokines (e.g., CCR2, CCR10) and tumor necrosis factor (TNF) signaling via NF-kB (e.g., TNFAIP3, NFKB1 and NFKB2) (Fig. 2C and Additional file 1: Table S2).Upregulation of the immune genes program was found in post-treated samples (Fig. 2D), suggesting therapy induced anti–myeloma immune response mediated by multiple pathways activation. Mapping malignancy-specific regulon networks by SCENIC [33], we also identified several key transcriptional factors (TFs) associated with treatment (Fig. 2E). For instance, XBP1 and STAT3 showed reduced regulon activities, in lined with decreased UPR and IL6-STAT3 signaling following treatment. On the contrary, FOSB, MYC and its translational regulator YBX1 exhibited increased regulon activities after treatment, therefore potentially supported anti-apoptosis activity and stress-response signaling under treatment pressure. Other TFs with upregulated activities associated with epigenetic regulation (EZH2, SMARCA4 and SETDB1) (Fig. 2E), indicating a chromatin remodeling during treatment. We also compared myeloma cells from responders who achieved VGPR/PR to tumor cells from non-responders who experienced SD (Additional file 1: Fig. S8A). Responders exhibited a significant reduction in cell proportion after treatment as expected (Additional file 1: Fig. S8B) and higher level of immune reactive score and related genes expression (e.g., HLA-A, HLA-E, HLA-F, CD74, CTSS) than non-responders (Additional file 1: Fig. S8C, D). By comparing myeloma cells in pre-treated samples with post-treated samples both in responders and non-responders, we found genes and pathways involving TGFβ signaling and cell adhesion were downregulated after treatment, while stress-associated genes and pathways (e.g., apoptosis, reactive oxygen species) showed upregulated after treatment both in responders and non-responders (Additional file 1: Fig. S8E, F).

Taken together, we demonstrated dynamic transcriptional programming in myeloma cells and identified four distinct cancer programs that affected by VCD therapy.

### Low immune reactive program predicted unfavorable drug response and prognosis in MM

**Fig. 3:**
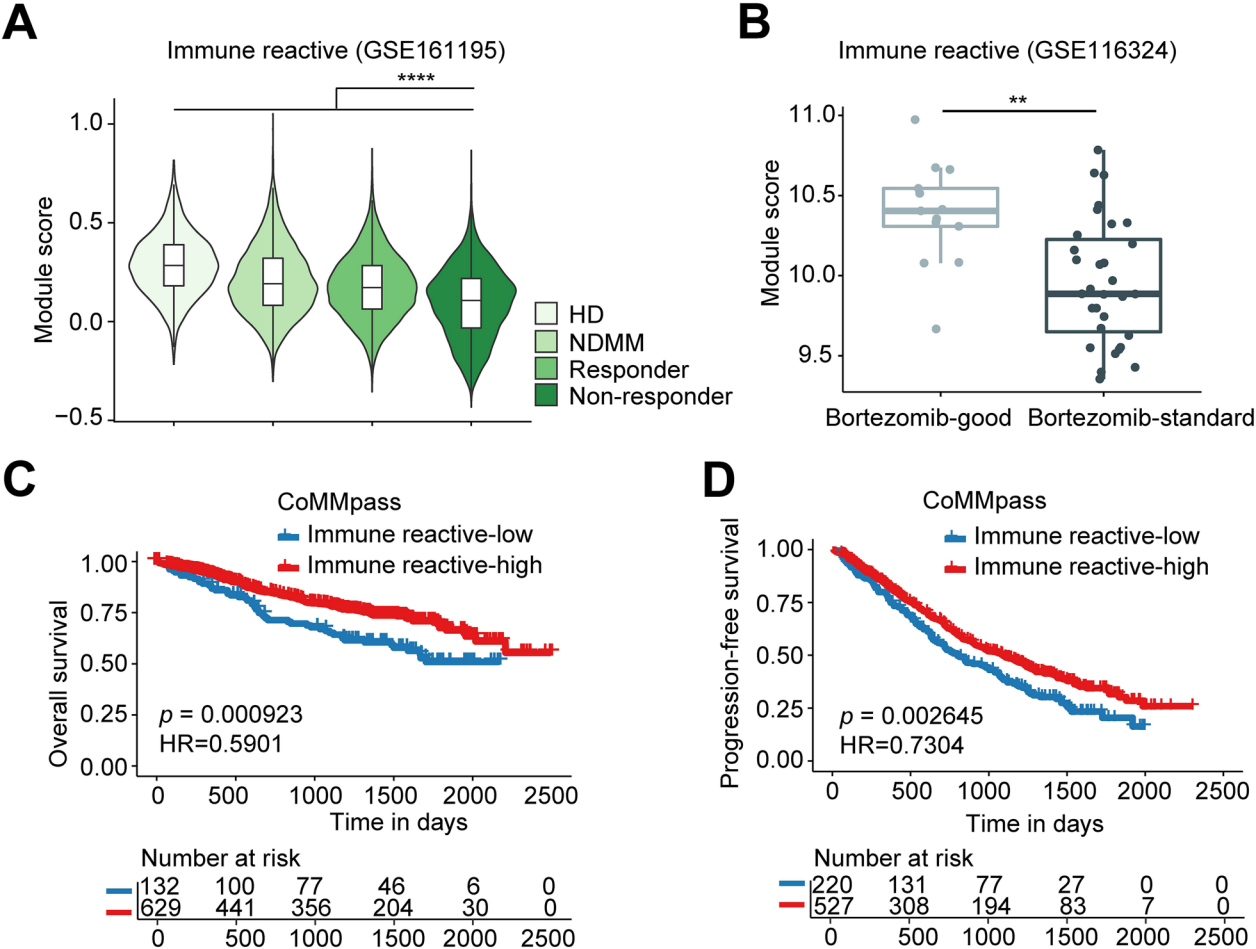
Immune reactive program predicted drug response and clinical outcome of patients with MM. **A** Violin plot of immune reactive score in patients from GSE161195 dataset. **B** Box plot of immune response score in MM patients from GSE116324 dataset (Bortezomib-good, n = 13; Bortezomib-standard, n = 31). **C**, **D** KM plots and analysis for OS (**C**) and PFS (**D**) comparing NDMM patients in CoMMpass data with high immune reactive score (red) and patients with low immune reactive score (blue). Statistical analysis was performed by Wilcoxon test, **p < 0.01, ****p < 0.0001. *KM* Kaplan Meier

The increase in stress and immune reactive programs following treatment prompted us to investigate whether they play roles in disease progression and resistance to therapy in large cohorts. By scoring NDMM samples in CoMMpass cohort, we found limited impacts of stress program in patient prognosis (Additional file 1: Fig. S9A, B), therefore we focused on the immune reactive signature. We examined scRNA-seq data from KYDAR study (GSE161195) [34] and found that immune reactive score was significant lower in non-responders compared with responders who received daratumumab and carfilzomib-based combined regimens (Fig. 3A). We also re-analyzed the RNA-seq data from another independent study (PADIMAC, GSE116324) [35] that denoted a bortezomib-good group who achieved VGPR or better and progression free at 1 year without autologous stem cell transplantation (ASCT), and remaining MM defined as bortezomib-standard group. By scoring tumor cells from this study, we found that immune reactive score was significant lower in bortezomib-standard patients compared with bortezomib-good patients (Fig. 3B), indicating low immune response may compromise drug sensitivity. We then further tested whether this signature can affect patient prognosis, and found that low immune reactive score was significantly associated with worse OS and PFS (Fig. 3C, D). These findings collectively suggested that the immune-reactive program was linked to unfavorable drug response and thereby conferring poor outcome to patients whose tumor possessed low level of such cell state.

### Tumor cells with lower immune reactive program exhibited immune escape phenotype

**Fig. 4:**
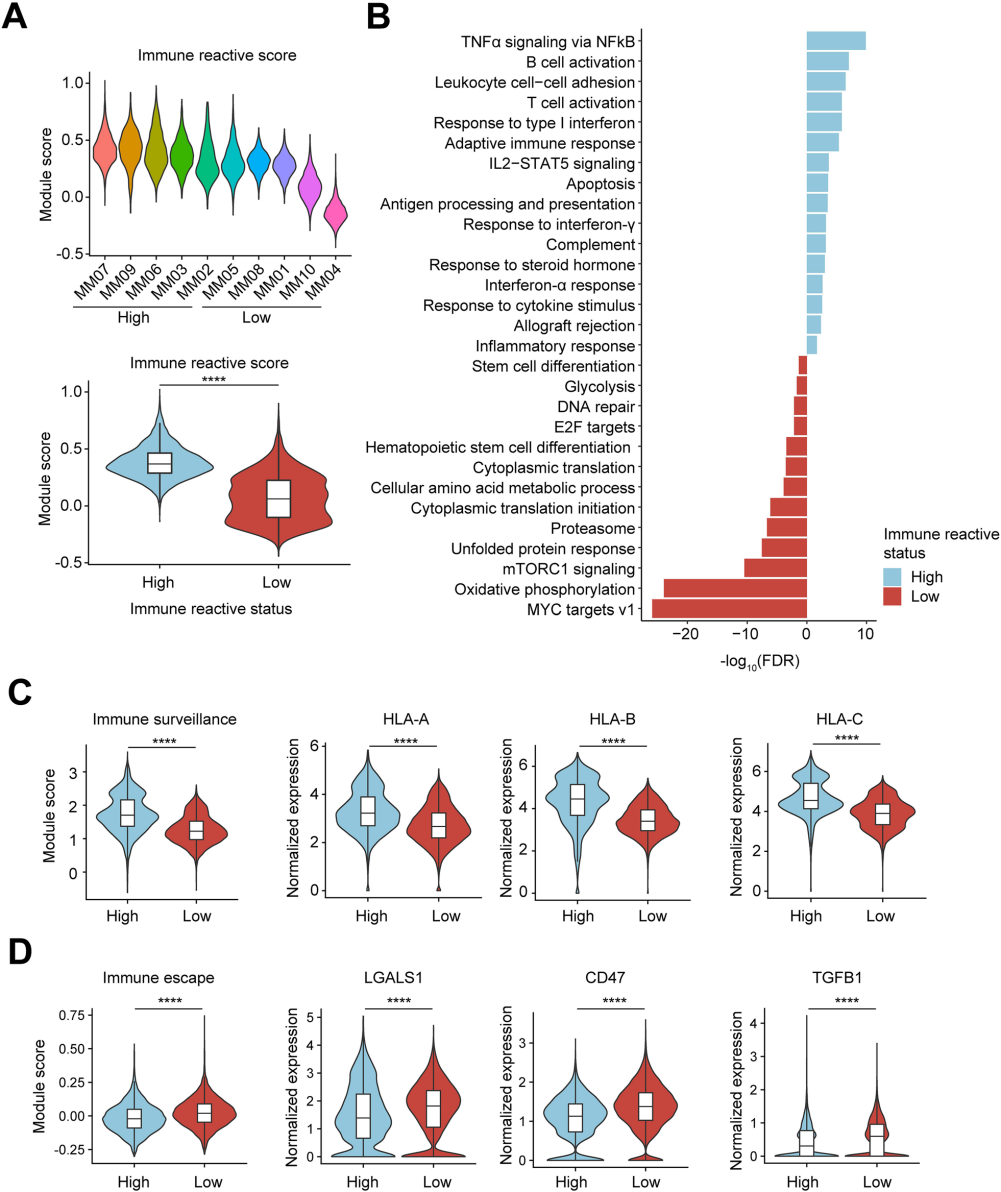
Differential expression analysis of tumor cells with different immune reactive scores. **A** Violin plot showing immune reactive score in each MM (up) and patients were divided into high or low status group (bottom). **B** Bar chart showing the enrichment of specific pathways, based on the HALLMARK, GO-BP and KEGG gene sets of upregulated and downregulated genes in high-immune reactive status samples compared with low status samples. **C** Violin plots showing immune surveillance score and expressions of MHC class I molecules. **D** Violin plots showing immune escape score and expressions of immunosuppressive genes. Statistical analysis in **A**, **C**, **D** was performed by Wilcoxon test, ****p < 0.0001. High, n = 5; Low, n = 5

After observing the impacts of immune reactive signature on MM clinic outcome, we next explored the transcriptional profiles of tumor cells with discrete immune reactive score and identified their differences that potentially contributed to unfavorable prognosis. We firstly determined the expression score of immune reactive genes in tumor cells from each patient and separated them into a high or low group according to their scores (Fig. 4A). We then performed pathway analysis comparing the two groups, and found that tumors with low immune reactive score possessed downregulation of various immune response pathways as expected, including TNFα and IFN signaling, whereas showed upregulation of transcriptional networks related to cell division, MYC signaling and metabolic process (Fig. 4B). To further confirm this “immune-exclusion” phonotype, we examined the expression of genes regulating immune surveillance and demonstrated significant decreased expressions of MHC class I molecules, thereby, impaired immune surveillance in low-immune reactive tumors (Fig. 4C). We also determined the expression of immune suppressive genes, and observed an increase in immune escape score and high expressions of CD47, LGALS1 and TGFB1 in low-immune response myeloma (Fig. 4D). These data suggested that these tumors possessed active cell growth and proliferation as well as high capacity to evade immune-mediated cell death.

### Characterization of dysfunctional immune cells in tumor microenvironment

We next explored the changes of major populations of immune cells pre/post-treatment. We characterized DEGs (Additional file 2) and differential pathways (Additional file 1: Fig. S10A, B) between pre- and post-treatment samples in each cell type**.** Among those, IFN-response genes, such as IFI44L, MX1, IFI6, ISG15, stress-associated genes, such as DDIT4, FOS, JUNB, and related signaling pathways were downregulated in most of immune cells after treatment. Whereas, histone genes, such as HIST1H1E, HIST1H1D, HIST2H2AC, HIST1H4C showed common upregulations in immune cells after treatment. In addition, cytotoxic markers such as NKG7 and GZMB in T cells and proinflammatory genes including S100A8, IRF1, CCL3, CCL3L1, TNFSF13B in myeloid subsets showed upregulations after treatment (Additional file 1: Fig. S10A, B, Additional file 2).

**Fig. 5:**
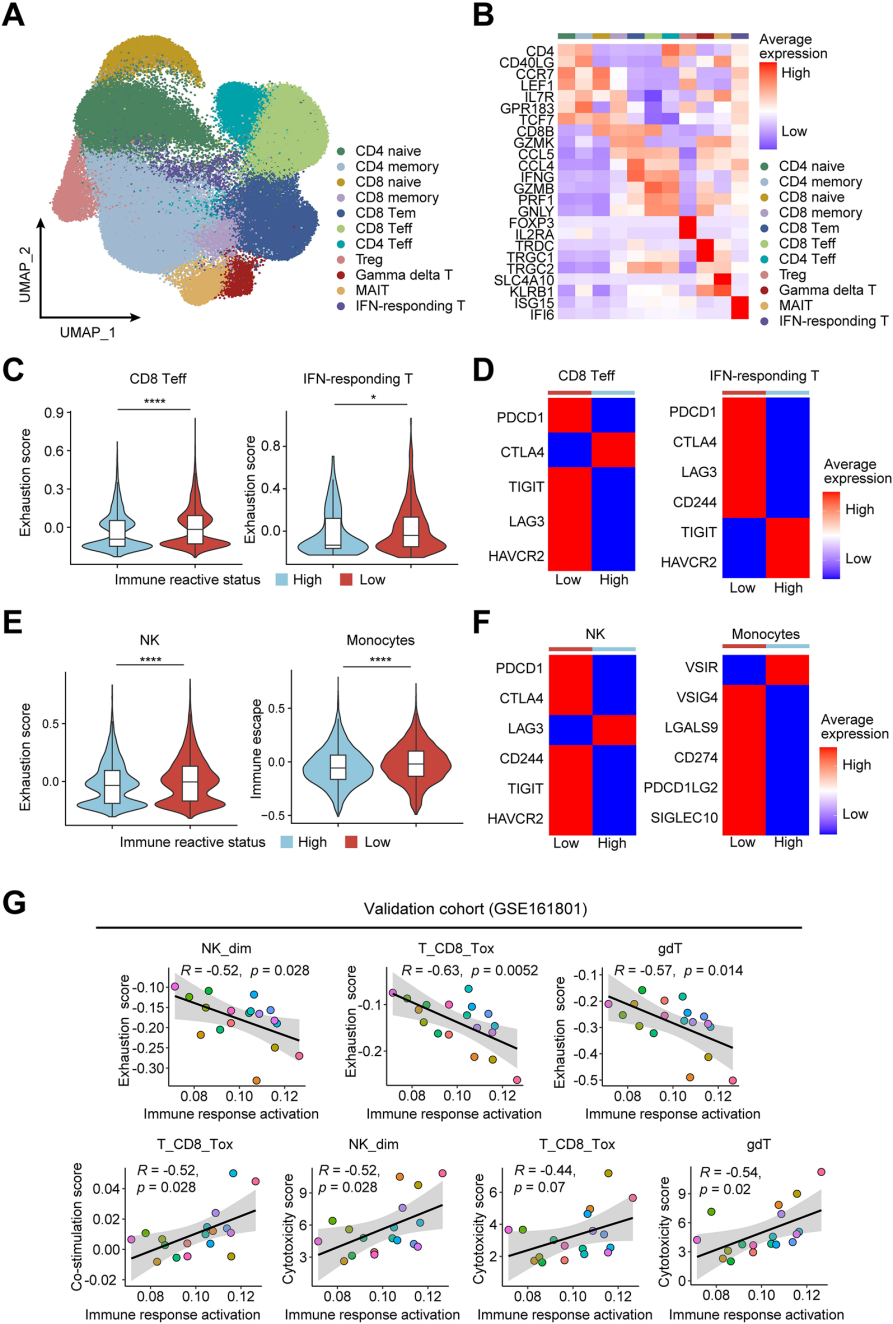
Functional analysis of immune cells in TME. **A** UMAP plot showing the re-clustering of T cell subsets colored by cell type. **B** Heat map showing averaged gene expression level of T cell subsets marker genes. **C** Violin plots showing exhaustion scores of CD8^+^ effector T cells (left) and IFN-responding T cells (right) in high/low immune reactive tumors. **D** Heat maps of average gene expression levels of immune checkpoints in CD8^+^ effector T cells (left) and IFN-responding T cells (right). **E** Violin plots showing exhaustion scores of NK cells (left) and immune escape scores in monocytes (right) in high/low immune reactive tumors. **F** Heat maps of average gene expression levels of immune checkpoints and immunosuppressive genes in NK cells (left) and monocytes (right). **G** Scatterplots showing pearson correlations between immune response activation with scores of co-stimulation, cytotoxicity and exhaustion per patient in GSE161801 dataset. Statistical analysis in **C**, **E** was performed by Wilcoxon test, *p < 0.05, ****p < 0.0001

Based on the observation of immune escape phenotype above, we then further delineated the relationship between immune-associated state in cancer cells and immune cell composition and function in tumor microenvironment (TME). While except for immature B cells, none of the major immune cell types showed substantial proportional differences between patients with immune-reactive high and low tumors (Additional file 1: Fig. S11), we then analyzed functional differences in the most prevalent immune cell types. We firstly extracted T cells in MM samples identifying 11 subsets in all T cells (Fig. 5A, B), and examined changes associated with their functional states. CD8^+^ effector T and IFN-responding T cells together with NK cells displayed significant increased exhaustion scores in tumors with low immune reactive status compared with high-immune response tumors, evidenced by higher expression of immune checkpoint receptors including PD-1 (PDCD1), LAG3 and TIM3 (HAVCR2) (Fig. 5C–F). Additionally, monocytes also showed increased expression of immune checkpoints and evasion genes in these patients (Fig. 5E, F). To verify the relationship between tumor intrinsic immune reactive signaling and T/NK cell function, we extracted myeloma cells from RRMM patients with paired immune cells in these patients (GSE161801) [9], and analyzed the signatures identified in our dataset. Results showed that signature of immune response activation in myeloma cells was positively correlated with co-stimulation score in CD8^+^ cytotoxic T cells (T_CD8_tox), cytotoxicity score in NK^dim^, CD8^+^ effector memory T and γδT cells (gdT) (Fig. 5G). In contrast, immune response activation in tumor cells was negatively correlated with exhaustion score in CD8^+^ cytotoxic T, NK^dim^ and γδT cells (Fig. 5G). These data suggested that early reduction of immune activation in tumor persisted in relapse stage, and compromised function of CD8^+^ effector T, NK and γδT cells in immune exclusion TME contributed to myeloma cells immune evasion.

Cellular interactions of tumor and BM microenvironment (BME) cells mediated by specific ligands and receptors affect disease progression and treatment resistance [36]. Therefore, we further predicted cellular interactions based on the expression of ligand-receptor pairs. Most pronounced interactions were found between myeloma cells and monocytes and dendritic cells (Additional file 1: Fig. S12A), in line with previous observations in RRMM [9]. Notably, several T cell and myeloid subsets showed lower tendency of interactions with myeloma cells in patients with low immune reactive compared with the high group (Additional file 1: Fig. S12A). By investigating individual interactions, we observed a frequent downregulation of pair of genes in immunomodulation in patients with low immune reactive, including CD86-CD28, TNFRSF10A-TNFSF10, CD74-APP/COPA/MIF, HLA-C-FAM3C and HLA-E-NKG2C (KLRC2), while upregulated pro-myeloma gene pairs including TGFB1-TGFBR2/TGFBR3, LGALS9-CD44 and CD47-SIRPA (Additional file 1: Fig. S12B).

### YBX1 was involved in immune response, immune cells infiltration and clinic outcome in MM

**Fig. 6:**
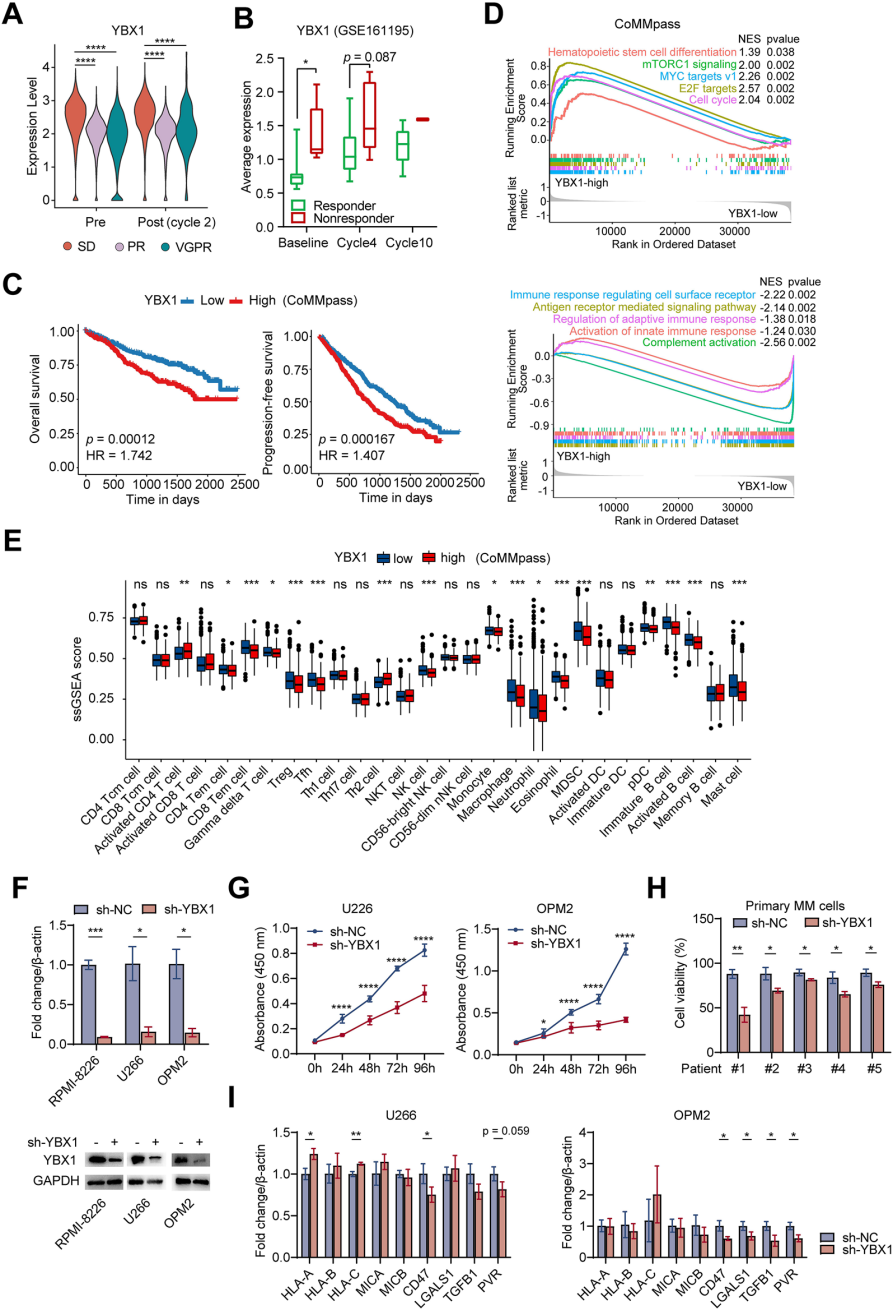
Correlations of YBX1 expression with drug response, immune cells infiltration and clinic outcome. **A** Violin plot of YBX1 expression in MM patients with different drug response pre and 2 cycles post treatment. **B** Box plot showing YBX1 expression in responders and non-responders at different timepoints from GSE161195 dataset. **C** KM plots and analysis for OS (left**,** logrank test, two-sided *p* = 0.00012, HR = 1.742) and PFS (right**,** logrank test, two-sided *p* = 0.000167, HR = 1.407) comparing NDMM patients in CoMMpass dataset with low YBX1 (blue) and high YBX1 expression (red). **D** GSEA analysis showing significant positive enrichment (top) and negative enrichment (bottom) of biological processes and signaling pathways in tumor cells from the YBX1-high group. **E** The differential score of 28 immune cell type signatures by ssGSEA method in patients from CoMMpass database with low and high YBX1 expression. **F** qPCR (up) and western blot (bottom) analysis showing the mRNA and protein levels of YBX1 in HMCLs respectively after transduction of sh-YBX1 or sh-NC. **G** Proliferation curves of U266 (left) and OPM2 (right) cell lines with transduction of sh-YBX1 or sh-NC using CCK-8 assay. **H** Viability of CD138-positive primary myeloma cells 96 h after transfection with YBX1 shRNA. **I** qPCR analysis showing the mRNA expression of key targets in U266 (left) and OPM2 (right) cell lines after transduction of sh-YBX1 or sh-NC. Error bars in **F**–**I** denoted mean ± SD. Statistical analyses in **A**, **B**, **E** were performed by Wilcoxon test and by two-tailed Student *t* test in **F**–**I**. ns not significant, *p < 0.05, **p < 0.01, ***p < 0.001, ****p < 0.0001

Based on the comprehensive alterations in cellular programs of tumor and microenvironment under treatment, we hypothesized that specific gene expression changes in myeloma cells can modulate tumor signaling and regulate immune response to TME. Notably, we identified a transcription factor YBX1 (Y-box binding protein-1, YB-1), which was upregulated in myeloma cells with low immune reactive status (Additional file 1: Fig. S13A), and showed negative correlation with MHC class I molecules expression, whereas positive correlation with expressions of immunosuppressive genes including LGALS1 and TGFB1 (Additional file 1: Fig. S13B). These data indicated that YBX1 might represent a potential factor that regulated the immune response program. Additionally, YBX1 showed upregulation in tumors from SD patients compared with patients of VGPR/PR both at baseline and 2 cycles of treatment (Fig. 6A). Through longitudinal analysis on primary refractory MM (PRMM) patients from GSE161195 dataset [34], we validated the correlation between high YBX1 expression and inferior drug response (Fig. 6B). Survival analyses further revealed high YBX1 expression significantly predicted poor OS and PFS in MM patients (Fig. 6C). Consistently, gene set enrichment analysis (GSEA) on CoMMpass cohort revealed that multiple biological processes, including hematopoietic stem cell differentiation, mTORC1 signaling, MYC target, cell cycle were upregulated in MM highly expressed YBX1 compared with low-YBX1 group (Fig. 6D). In contrast, pathways involving activation of immune response were downregulated in YBX-high group (Fig. 6D). These resembling features from low immune-reactive patients identified above led us to test impacts of YBX1 expression on TME composition. By estimating immune cells infiltration in CoMMpass cohort using ssGSEA method, we observed that most of the TME components, such as CD4^+^/CD8^+^ Tem, γδT, NK subsets, multiple myeloid subsets and B cells were significant lower in patients with high YBX1 expression compared with those with low YBX1 (Fig. 6E). Collectively, these results suggested that high level of YBX1 may confer “cold” tumor immune phenotype, characterized by reduced abundance of infiltrating leukocytes, diminished immune cell activation and emergence of immunosuppressive signaling, which might represent a promising therapeutic target.

To validate the findings from our scRNA-seq data regarding YBX1 function in MM, we further performed in vitro experiments on human myeloma cell lines (HMCLs) and primary myeloma samples. After the knockdown of YBX1 (Fig. 6F), cell proliferation was significantly decreased in U266, OPM2 (Fig. 6G) and RPMI-8226 (Additional file 1: Fig. S14A) myeloma cell lines. Consistently, reduced cell viability was found in primary myeloma cells after YBX1 suppression (Fig. 6H). To further explore the potential mechanisms of YBX1, we tested several key genes involving immune response identified in scRNA-seq analysis. The qPCR results showed that knockdown of YBX1 upregulated immune surveillance genes including HLA-A, HLA-C, MICB, while downregulated immune suppressive genes including CD47, LGALS1 and PVR (ligand for TIGIT) in U266, OPM2 (Fig. 6I) and LGALS9 (ligand of TIM-3) in RPMI-8226 (Additional file 1: Fig. S14B, C). Tumor-T cell interaction is required for T cells to recognize and eliminate cancer cells. We further examined the myeloma-T interactions with YBX1 knockdown. Through co-culture of T cells and myeloma cells from RPMI-8226 cell line, we found that tumor-binding T cells were increased after YBX1 knockdown in RPMI-8226, which indicated enhanced myeloma-T cells interaction when reducing YBX1 expression (Additional file 1: Fig. S14D, E). To further investigate the effect of co-culture on T cell function, we determined the expression of several exhausted and cytotoxic markers of gated CD8^+^ T cells (Additional file 1: Fig. S15A). When co-cocultured with RPMI-8226 myeloma cells with YBX1 knockdown, the frequencies of IFN-γ^+^ and GZMB^+^ cells among CD8^+^ T cells were significantly higher than those in CD8^+^ T cells co-cultured with control RPMI-8226 cells (Additional file 1: Fig. S15B, C). On the contrary, the frequency of TIM3^+^ CD8^+^ T cells showed a significant decrease after knocking down YBX1 in myeloma cells (Additional file 1: Fig. S15D), which was consistent with the decreased expression of LGALS9 in RPMI-8226 with YBX1 knockdown (Additional file 1: Fig. S14C), whereas other exhausted markers remained unchanged (Additional file 1: Fig. S15E). These data suggested that inhibition of YBX1 in myeloma cells promoted recovery of CD8^+^ T function.

Together, these results recapitulated and validated the gene expression patterns observed in our scRNA-seq analysis, and suggested that YBX1 facilitated its impacts on myeloma survival and drug response by affecting T cells function and promoting immune suppression.

## Discussion

Despite the intensive improvement in developing new agents, MM remains incurable. Therefore, comprehensive understanding of pathogenesis and drug resistance is of major importance. In this study, we applied scRNA-seq on MM patients before and after treatment, and identified a distinct tumor program associated with immune response that regulated tumor signaling and TME alteration, therefore influenced drug response and patient’s survival.

Analyzing data at single-cell resolution helps us to precisely detect both integral and detailed complexities in genomic and transcriptional profiles in cancer cells [9, 37, 38]. Our scRNA-seq data revealed widespread inter- and intra-tumor heterogeneity in myeloma cells, which provides a potential explanation for differential responses to the same therapy due to genomic and transcriptional complexity. We further demonstrated the significant correlation between ITH and drug resistant and patient prognosis in MM, highlighting potential values of ITH in clinical practice [39–41].

We showed predominant treatment-induced transcriptional reprogramming in myeloma cells, characterized by expression changes of four distinct tumor cell signatures. Increased expression of the immune reactive program after treatment may indicate that systemic tumor immunity was induced by VCD therapy, which was consistent with the findings in previous study [42]. However, MM with low immune response signature was linked to poor clinical outcome, and together with dysfunctional features in their immune compartments. These findings imply co-evolution of myeloma cells and surrounding immune components during treatment. In particular, these data suggested compromised immune activation might be induced by impairment of MHC class I/IFN-mediated immunosurveillance, which supporting the observations from triple-refractory MM patients [43], and indicated that under treatment pressure, myeloma cells responded at an early timepoint to escape from immune system for its survival and subsequent resistance. Therefore, our analysis demonstrated the alteration of immune reactive program as early as 2 cycles after starting treatment, and provide important information for later drug response and survival of MM patients, which may represent an early predictor. And targeting this tumor program could be important, especially when combining chemotherapy with immunotherapy to help boost anti-myeloma immune response.

Y-box binding protein 1 (YB-1), encoded by YBX1 gene, belongs to the family of DNA/RNA binding proteins with a highly conserved cold-shock domain [44]. Many reports point to YBX1 as a regulator of cellular proliferation, tumor metastasis and a determinant of cancer stem cell function in multiple cancer types [45–49]. In particular, YBX1 has been found contributing to disease progression, survival, and drug resistance in MM [50], probably through MYC/YBX1 oncogenic circuit [51, 52]. Given that myeloma cell oncogenesis and the BME are tightly linked, strategies that target both compartments appear to be particularly important. Our data suggested YBX1 may contribute to myeloma cells proliferation, as well as immune modulation, which indicate potential roles of YBX1 in regulating both tumor-intrinsic programs and microenvironment remodeling. Notably, our in vitro results indicated that dysregulation of immune genes including MHC class I molecules and immunosuppressive genes may serve as a novel mechanism of YBX1 in regulating immune escape in myeloma, which is similar to the finding that YBX1 signaling contributed to tumor immune evasion and resistance by programmed death-1 ligand 1 (PD-L1) in hepatocellular carcinoma [53]. Hence, YBX1 represents a promising target for MM, which can be evaluated in detail for future investigation.

The study reported herein has several limitations. First, single-cell sequencing is limited in few patients, which prevents us to access comprehensive overview and broadly inspect myeloma pathogenesis and drug resistance. Further investigation in large-scale MM samples is necessary. Second, our present study discovered some interesting changes of both myeloma cells and immune cells including T cells, NK cells and monocytes during treatment, while further experiments including in vitro or in vivo functional validation experiments, are required to address the underlying mechanisms.

## Conclusions

In summary, our results reveal a systematic landscape of heterogeneous malignancy and microenvironmental changes of MM at the single-cell resolution and identified specific transcriptomic programs based on dynamic profiling of pre- and post-treatment. These findings shed light on the molecular and cellular complexity of MM during treatment and provide potential molecular biomarkers for drug response and therapeutic options.

## Methods

### Patients and samples

Ten patients who were pathologically diagnosed with active MM, and 3 age-, sex-matched healthy volunteers were enrolled in this study. Their demographic characteristics are summarized in Additional file 1: Table S1. Fresh specimens of bone marrow mononuclear cells (BMMC) and matched peripheral blood mononuclear cells (PBMC) from MM patients and healthy volunteers were collected. In total, 10 BMMC and matched PBMC were obtained in MM patients before treatment, but due to one patient (MM09) who died from disease, 9 BMMC and matched PBMC were obtained after 2 cycles of VCD treatment. 3 BMMC and matched PBMC samples from healthy volunteers were harvested. All MM patients received the induction therapy of VCD regimen, and drug response was evaluated according to the International Myeloma Working Group (IMWG) consensus criteria [28]. All patients were given informed consent for collection of clinical information, sample collection, research testing under the Ethics Committee-approved protocols (2019010) at Renji Hospital Affiliated to Shanghai Jiao Tong University School of Medicine.

### Tissue acquisition and cell preparation

Fresh BM and PB samples were obtained and processed immediately upon receipt and were diluted with chilled phosphate-buffered saline (PBS, Gibco) and carefully layered over Ficoll-Paque PLUS (Cytiva, Sweden), and then centrifuged at 2000 rpm for 20 min at 20 °C without brake. Mononuclear cells at the middle layer were carefully transferred and washed twice with PBS, and then cell pellets were resuspended in 1 ml PBS + 0.04% BSA. Cell suspensions were counted with TC20 automated cell counter (Bio-Rad) to determine cell concentration and viability.

### Droplet-based single-cell sequencing

According to the manufacturer’s protocol, Chromium Single cell 3′ Reagent v3 kits (10× Genomics) were used to prepare barcoded scRNA-seq libraries. Single-cell suspensions were loaded onto a Chromium Single-Cell Controller Instrument to generate single-cell gel beads in emulsions (GEMs). After generation of GEMs, reverse transcription reactions were engaged to generate barcoded full-length cDNA, which was followed by disruption of emulsions using the recovery agent, and then cDNA clean-up was performed with DynaBeads Myone Silane Beads (Thermo Fisher Scientific). Next, cDNA was amplified by PCR for the appropriate number of cycles, which depended on the number of recovered cells. Subsequently, the amplified cDNA was fragmented, end-repaired, A-tailed, and ligated to an index adaptor, and then the library was amplified. Every library was sequenced on a Novoseq 6000 platform (Illumina), and 150 bp paired-end reads were generated.

### Raw data processing and quality control

Cell Ranger (version 2.2.0) was used with default parameters to process the raw data, and generate gene expression matrix per cell. Ambient RNA signal was removed using the default SoupX [54] (v1.4.5) workflow. Samples were then converted into a Seurat object by the R package Seurat [55] (version 3.2.0). The low-quality cells were filtered: such as cells with mitochondrial counts > 15% and nFeature_RNA < 200. DoubletFinder [56] (v2.0) was then used to identify putative doublets in each sample individually, and the number of expected doublets was calculated for each sample based on the expected rates of doublets which are provided by 10× Genomics. Finally, 241,440 single cells remained, and applied in downstream analyses.

We used Seurat to perform standard library size and log-normalization, and FindVariableFeatures function was performed to detect highly variable genes (HVGs) and 2000 HVGs were selected. To remove batch effects, log-normalized counts for each batch were used as input to the RunFastMNN function from SeuratWrappers package with default parameters. The results of RunFastMNN [24] were projected to uniform manifold approximation and projection (UMAP), then a shared nearest-neighbor network was created based on Euclidian distances between cells in 30 principal component spaces. The main cell clusters were identified with the FindClusters function by Seurat with resolution set to 0.8, and visualized with UMAP plots.

### Identification of DEGs and cell type annotation

To identify DEGs in each cell types, the FindAllMarkers function of the Seurat with the following parameters was used: logFC threshold, 0.25 and adjusted *P* value < 0.05. The ‘MAST’ test was used for DEG analysis. Plasma cells were identified based on expressing high levels of TNFRSF17, SDC1, and SLAMF7. Other cell types were annotated using classical immune cell marker expression according to the description given in Additional file 1: Table S2.

### Reclustering of the cell subtypes

To identify subclusters within cell subtypes, we reselected the HVGs for each cell subtype as described above and then applied dimensionality reduction. Batch effect correction and UMAP dimensionality reduction using default and graph-based clustering cell reclustering were also performed as described above. For plasma cell subset, immunoglobulin genes were removed before the reclustering.

### Estimation of CNAs (copy number alterations) in plasma cells

The inferCNV [26] package was used to detect the CNAs in individual plasma/myeloma cells and to recognize real cancer cells with default parameters. As reference, we used normal plasma cells (nPCs) derived from the healthy donors and profiled CNAs in myeloma cells of every patient individually.

### SCENIC (single-cell regulatory network inference and clustering) analysis

The SCENIC analysis was run as described in Aibar et al. (2017) [33], using the R SCENIC package (version 1.1.2-2) and hg19-500 bp-upstream-10species databases for RcisTarget, GRNboost, and AUCell. The input matrix was the normalized expression matrix from Seurat.

### Pathway analysis and definition of signature scores

Differential expression analysis comparing myeloma cells pre- and post-treatment was performed using the FindMarker function provided by Seurat. LogFC threshold, 0.25 and adj.p.val < 0.05 were used as the cut-off criteria. Enrichment analysis based on Molecular Signatures Database (MsigDB, https://www.gsea-msigdb.org/gsea/index.jsp) hallmarks, oncology (C5:BP) and KEGG (C2:KEGG) gene sets were performed on these DEGs with ClusterProfiler. Signature scores were calculated by AddModuleScore function from Seurat R package. Signature gene lists were derived from MsigDB or selected gene sets curated from literature (Additional file 1: Table S2).

### Cell–cell communication analysis

Cellular communication analysis was performed using CellPhoneDB [57] Python package (v2.0) with default settings.

### Cell culture

Human myeloma cell lines (HMCLs) RPMI-8226, U266, OPM2 were cultured in RPMI-1640 medium (Gibco/Thermo Fischer Scientific, USA) with 10% fetal bovine serum (FBS, Gibco/Thermo Fischer Scientific, USA) and 1% Penicillin/Streptomycin solution (Gibco/Thermo Fischer Scientific, USA), and incubated in a humidified incubator at 37 °C and 5% CO_2_ atmosphere.

### shRNA lentivirus production and infection

shRNA-encoding plasmids were obtained from the Thermo Scientific Open Biosystems GIPZ Lentiviral shRNA Library. The hairpin vectors were co-transfected with the lentivirus expression plasmid and packaging plasmid into actively growing HEK293FT cells using jetPRIME transfection reagent (Polyplus, USA) according to the manufacturer’s instructions. Virus containing supernatants were collected at 48 h after transfection and filtered through 0.45 μm cellulose acetate filters. HMCL cells were plated in 6-well plates at 20–30% confluence and infected for 12 h in the presence of 8 μg/ml polybrene. And the infection was repeat twice. After infection, the cells replaced with fresh media. Knockdown efficiency was confirmed by qPCR or western blot. YBX1 shRNA target sequence: 5′-CCAGCAAAATTACCAGAAT-3′.

### Western blot

Cells were lysed in RIPA with protease inhibitor cocktail (Roche) and loaded per lane onto 10–12% SDS PAGE gels. After transfer, PVDF membranes were blocked and incubated overnight at 4 °C with primary antibody. After three washes in TBST, membranes were incubated for 1 h at room temperature with horseradish peroxidase (HRP)-conjugated anti-rabbit secondary antibodies (#7074, CST), then washed a further three times with TBST. Then immunoreactive protein bands were imaged using Immobilon Western Chemiluminescent HRP Substrate (Millipore). Primary antibodies used were as follows: YBX1 (#4202, CST), GAPDH (#2118, CST).

### RNA isolation and qPCR

Total RNA was extracted using the Trizol (Invitrogen, USA) according to the manufacturer’s instructions.

Total RNA (1 μg) was reverse transcribed using HiScript® III Reverse Transcription kit (Vazyme, China). Each cDNA sample was analyzed in triplicate with the CFX Connect Real-Time PCR Detection System (Bio-Rad, USA) using ChamQ Universal SYBR qPCR Master Mix (Vazyme, China). The primer sequences were listed in Additional file 1: Table S3.

### Cell proliferation and viability assay

Cells were seeded into 96-well plate (5000 cells/well) with 100 μL complete medium. We added 10 μL Cell Counting Kit-8 (CCK-8; Dojindo, Japan) to the wells at 24 h, 48 h, 72 h and 96 h, respectively, to detect the proliferation of cells. Then, we incubated cells in the incubator for 2 h at 37 °C out of light. We detected absorbance of each well at a wavelength of 450 nm using Multiskan GO Microplate Spectrophotometer (Thermo Scientific, USA) and calculated the cell proliferation rate. Cell viability was determined with 0.4% Trypan blue (Sigma-Aldrich, Canada) staining and calculated from the following formula: Percent cell viability equals the number of unstained (living) cells divided by the total number of cells times 100 [58].

### Human primary MM samples

Bone marrow aspirates were obtained from NDMM patients with written informed consent after approval of Ethics Committee at Ren Ji Hospital, Shanghai Jiao Tong University School of Medicine in accordance with the Declaration of Helsinki. Mononuclear cells were isolated from samples by Ficoll-Paque PLUS (GE Healthcare, USA). MM cells were purified using CD138 microbeads according to the manufacturer’s instructions (Miltenyi Biotech, Germany), and then used for in vitro experiments.

### Isolation of human primary T cells and co-culture with myeloma cell line

PBMCs derived from four healthy volunteers were isolated using a Ficoll‑Paque gradient. T cells were purified from PBMCs using CD3 microbeads according to the manufacturer’s instructions (Miltenyi Biotech, Germany), and maintained in RPMI-1640 supplemented with 10% FBS, 1% Penicillin/Streptomycin solution, and 300 units/ml of recombinant human IL-2 (Peprotech, USA). 1 × 10^5^ RPMI-8226 tumor cells with 2 × 10^5^ T cells were seed and co-cultured in 48-well plate. After 12 h of co-culture, microphotograph images in brightfield were captured and the number of T cells binding to myeloma cells were manually counted. For the quantification analysis of Tumor-T interaction, 15 fields in each group (sh-NC and sh-YBX1) were selected to measure the percentage of binding T cell to myeloma in total cells, and averaged binding of T cells from four healthy volunteers was calculated. For T cell function analysis, PBMCs derived from 7 healthy volunteers were isolated using a Ficoll‑Paque gradient and T cells were further purified using CD3 microbeads. Then CD3^+^ T cells were stimulated with anti-CD3/CD28 microbeads (#11131D, ThermoFisher), and co-cultured with RPMI-8226 tumor cells in 48-well plate with a ratio of Tumor:T = 1:2 (1 × 10^5^ tumor cells with 2 × 10^5^ T cells). After 5 days of co-culture, cells were harvested and subjected to subsequent flow cytometric measurements.

### Flow cytometry analysis

The expression of exhausted and cytotoxic markers of T cell was analyzed by flow cytometry. After 5 days of co-culture, cells were suspended in PBS containing 2% FBS and incubated according to the manufacturer’s instructions with the following fluorochrome-labeled antibodies: anti-CD8-APC-A700 (#B49181, Beckman Coulter), anti-CD4-APC (#IM2468, Beckman Coulter), anti-TIGIT-PE-Cy7 (#372714, Biolegend), anti-CTLA-4-BV785 (#369624, Biolegend), anti-Tim-3-PE-Cy7 (#345052, Biolegend), anti-LAG-3-BV605 (#369324, Biolegend), anti-PD-1-BV510 (#367424, Biolegend), anti-Granzyme B-PE (#372208, Biolegend), anti-IFN-γ-FITC (#IM2716U, Beckman Coulter). Cells were stained with fluorochrome-conjugated antibodies for 30 min at room temperature in the dark. For intracellular staining, surface-stained cells were fixed and permeabilized using PerFix-nc Kit (#B31168, Beckman Coulter) according to the manufacturer's instructions. Flow cytometry analyses were performed on DxFlex system (Beckman Coulter) and data were analyzed using FlowJo software (v10.5.3).

### Statistical analysis

All statistical analyses and graph generation were performed in R (version 3.6.2) and GraphPad Prism (version 8.0). Significance was calculated using the indicated statistical tests.

## Supplementary Information

**Additional file 1: Table S1.** Characteristics of healthy donors and MM patients. **Table S2.** Gene signatures in tumor and immune cells. **Table S3.** qPCR primer sequences. **Figure S1.** Comparison of integration performance by different methods. **Figure. S2** Single cell assessment of MM and healthy donors. . **Figure S3.** Characterization of PCs from BM and PB. **Figure S4.** Copy number alterations (CNAs) analysis from scRNA-seq data. **Figure S5.** Characterization of nPCs from healthy donors and myeloma cells from MM. **Figure S6.** Intra-tumor heterogeneity of MM. **Figure S7.** Analysis of myeloma cells pre/post-treatment.  **Figure S8.** Analysis of myeloma cells in responders and non-responders. **Figure S9.** Impacts of stress program on prognosis of MM patients. **Figure S10.** Analysis of immune cells pre/post-treatment. **Figure S11.** Immune cell fractions. Box plot for the comparison of immune cell type fractions between immune reactive-high patients and low patients. **Figure S12.** Cellular interactions in MM with different immune reactive status. **Figure S13.** Correlations of YBX1 expression with immune response and escape in MM. **Figure S14.** RPMI-8226 cells proliferation curve, target genes expression and co-culture with T cells. **Figure S15.** Flow cytometric analysis of CD8^+^ T cells function co-cultured with RPMI-8226 cells.

**Additional file 2.** The list of DEGs comparing pre- and post-treatment samples in each immnue cell type.

## Data Availability

The raw scRNA-seq data that support the findings of this study are deposited in the China National Center for Bioinformation under accession code HRA001600. The IA14 release of CoMMpass data was downloaded from the MMRF researcher gateway portal (https://research.themmrf.org). Previously published scRNA-seq, bulk RNA-seq and microarray data that were reanalyzed in this study are available under following accession codes and links: GSE161195 (https://www.ncbi.nlm.nih.gov/geo/query/acc.cgi?acc=GSE161195), GSE161801 (https://www.ncbi.nlm.nih.gov/geo/query/acc.cgi?acc=GSE161801), GSE116324 (https://www.ncbi.nlm.nih.gov/geo/query/acc.cgi?acc=GSE116324).

## Abbreviations

MM: Multiple myeloma
scRNA-seq: Single-cell RNA sequencing
VCD: Bortezomib–cyclophosphamide–dexamethasone
PCs: Plasma cells
PIs: Proteasome inhibitors
IMiDs: Immunomodulatory drugs
ITH: Intra-tumor heterogeneity
NDMM: Newly diagnosis MM
HSPC: Hematopoietic stem and progenitor cell
GMP: Granulocyte–monocyte progenitor
nPCs: Normal plasma cells
IFN: Interferon
RRMM: Relapsed/refractory MM
NMF: Non-negative matrix factorization
CNA: Copy number alteration
OS: Overall survival
PFS: Progression-free survival
UPR: Unfolded protein response
TFs: Transcriptional factors
VGPR: Very good partial response
PR: Partial response
SD: Stable disease
ASCT: Autologous stem cell transplantation
BME: BM microenvironment
YBX1: Y-box binding protein-1
MHC: Major histocompatibility complex
GSVA: Gene set variation analysis
UMAP: Uniform manifold approximation and projection
HR: Hazard ratio
KM: Kaplan meier
HD: Healthy donor
ssGSEA: Single sample gene set enrichment analysis
*t*-SNE: *t*-Distributed stochastic neighbor embedding
ER stress: Endoplasmic reticulum stress
UMI: Unique molecular identifier
Meg: Megakaryocyte
Ery: Erythroid progenitors
DEGs: Differentially expressed genes
Btz: Bortezomib
FACS: Fluorescence-activated cell sorting
