# Supplementary material for: Dynamic single-cell RNA-seq analysis reveals distinct tumor program associated with microenvironmental remodeling and drug sensitivity in multiple myeloma
Source: Cell Biosci. 2023 Jan 30;13:19. doi: 10.1186/s13578-023-00971-2 (PMC9887807; doi:10.1186/s13578-023-00971-2)
Supplement: Supplementary file 1 — Additional file 1: Table S1. Characteristics of healthy donors and MM patients. Table S2. Gene signatures in tumor and immune cells. Table S3. qPCR primer sequences. Figure S1. Comparison of integration performance by different methods. Figure. S2 Single cell assessment of MM and healthy donors. . Figure S3. Characterization of PCs from BM and PB. Figure S4. Copy number alterations (CNAs) analysis from scRNA-seq data. Figure S5. Characterization of nPCs from healthy donors and myeloma cells from MM. Figure S6. Intra-tumor heterogeneity of MM. Figure S7. Analysis of myeloma cells pre/post-treatment. Figure S8. Analysis of myeloma cells in responders and non-responders. Figure S9. Impacts of stress program on prognosis of MM patients. Figure S10. Analysis of immune cells pre/post-treatment. Figure S11. Immune cell fractions. Box plot for the comparison of immune cell type fractions between immune reactive-high patients and low patients. Figure S12. Cellular interactions in MM with different immune reactive status. Figure S13. Correlations of YBX1 expression with immune response and escape in MM. Figure S14. RPMI-8226 cells proliferation curve, target genes expression and co-culture with T cells. Figure S15. Flow cytometric analysis of CD8+ T cells function co-cultured with RPMI-8226 cells. [file 13578_2023_971_MOESM1_ESM.docx]

**Additional** **information**

**Table S1. Characteristics of healthy donors and MM patients.**

| Sample ID | Age/Sex | FISH  abnormity | Therapy | Response^†^ | Timepoint ^‡^ | Sample collection | Group |
| --- | --- | --- | --- | --- | --- | --- | --- |
| MM01 | 41/M | t(11;14) | VCD | PR | Pre+Post | BM+PB | NDMM |
| MM02 | 53/F | t(11;14) | VCD | PR | Pre+Post | BM+PB | NDMM |
| MM03 | 57/F | t(4;14) | VCD | PR | Pre+Post | BM+PB | NDMM |
| MM04 | 51/M | Negative | VCD | SD | Pre+Post | BM+PB | NDMM |
| MM05 | 64/M | Negative | VCD | PR | Pre+Post | BM+PB | NDMM |
| MM06 | 51/M | t(6;14) | VCD | PR | Pre+Post | BM+PB | NDMM |
| MM07 | 63/F | t(4;14) | VCD | VGPR | Pre+Post | BM+PB | NDMM |
| MM08 | 63/M | t(4;14) | VCD | VGPR | Pre+Post | BM+PB | NDMM |
| MM09 | 63/F | Negative | VCD | NA | Pre | BM+PB | NDMM |
| MM10 | 50/F | t(4;14) | VCD | SD | Pre+Post | BM+PB | NDMM |
| HD01 | 56/F | - | - | - | - | BM+PB | Healthy donor |
| HD02 | 53/M | - | - | - | - | BM+PB | Healthy donor |
| HD03 | 50/M | - |  | - | - | BM+PB | Healthy donor |

^†^ Treatment response was assessed at 2 cycles of therapy by measuring monoclonal immunoglobulins and/or light chains in blood and urine in a clinical lab.

^‡^ Pre samples were collected at diagnosis, and post samples were collected after 2 cycles of therapy.

M, male; F, female.

| Gene signature | Gene |
| --- | --- |
| UPR | HSPA5, VCP, PMAIP1, GORASP2, SSR1, DNAJC3, DDIT4, NPM1, CCT8, HM13, SEC31A, DERL2, CREBRF, STT3B, SERINC3, DNAJA1, HSPA8, CALR, DNAJB9, RACK1, PDIA6, CDK5RAP3, ERP29, DDRGK1, BCAP31, SEC11A, MTHFD2, HSPA13, RNF187 |
| Metabolic-associated | SESN2, SLC3A2, SLC25A3, SLC7A11, SLC7A1, SLC38A10, SERINC3, CAPN2, SLC1A5, DPEP1, SLC44A1, SLC12A6, SLC25A39, SHMT2, ALDH1L2, PGP, PHGDH, PGAM1, ASNS |
| Stress-associated | PRDX4, ATOX1, FOS, JUN, JUNB, FOSB, EGR1, DDIT3, FTL, ROMO1, GSTP1, GCSH, ATF3, TP53INP1, MCL1, PARP1, CASP3, IER5, BCL2, DAD1, MDM4, GADD45A, PPP1R15A, BOD1L1, ASCC3, NFE2L1, NFE2L3 |
| Immune reactive | HLA-A, HLA-B, HLA-C, HLA-F, HLA-DOB, CD74, CTSS, XRCC5, PTPN6, HMGB1, CCR2, CCR10, FBXW11, IFIH1, ZBP1, IFITM1, IFNAR1, IFNAR2, IFITM2, IRF1, IRF2, IRF3, IRF4, NCOA3, SP100, BIRC3, TNFAIP3, NFKB1, NFKB2, IL2RG, NFKBIE, RELA, RELB, NFKBIA, PLEK, MALT1 |
| Immune response activation | GO_ACTIVATION_OF_IMMUNE_RESPONSE [1] |
| Co-stimulation | ICOS, CD226, TNFRSF12A, TNFRSF9, TNFSF14, TNFSF4, TNFRSF25, CD27, CD28, CD40LG, IL2RB |
| Cytotoxicity | PRF1, IFNG, FASLG, GZMA, GNLY, GZMH, GZMB, NKG7, CST7, TNFSF10 |
| Exhaustion | PDCD1, CTLA4, TIGIT, LAG3, HAVCR2, CD244 |
| Immune surveillance | HLA-A, HLA-B, HLA-C, MICA, MICB [2] |
| Immune escape | CD47, ADAM10, HLA-G, CD274, FASLG, CCL5, TGFB1, IL10, PTGER4 |

**Table S2. Gene signatures in tumor and immune cells.**

**Table S3. qPCR primer sequences.**

| Gene | Sequence 5’-3’ |
| --- | --- |
| YBX1-F | TGCAGCAGACCGTAACCATT |
| YBX1-R | TGGATCGGCTGCTTTTGTC |
| β-actin-F | CCACCATGTACCCTGGCATT |
| β-actin-R | ACTCCTGCTTGCTGATCCAC |
| HLA-A-F | CTCTTTGGAGCTGTGATCACT |
| HLA-A-R | GAAGGGCAGGAACAAMTCTTG |
| HLA-B-F | GTCCTAGCAGTTGTGGTCATC |
| HLA-B-R | TCAAGCTGTGAGAGACACATCA |
| HLA-C-F | TCCTGGCTGTCCTAGCTGTC |
| HLA-C-R | CAGGCTTTACAAGTGATGAGAG |
| MICA-F | TCAGAGTCATTGGCAGACAT |
| MICA-R | TGTGGCATCCCTGTGGTC |
| MICB-F | ACCTTGGCTATGAACGTCACA |
| MICB-R | CCCTCTGAGACCTCGCTGCA |
| CD47-F | GGCAATGACGAAGGAGGTTA |
| CD47-R | ATCCGGTGGTATGGATGAGA |
| LGALS1-F | CCTGGAGAGTGCCTTCGAGT |
| LGALS1-R | CACACGATGGTGTTGGCGTC |
| TGFB1-F | GAGGGGAAATTGAGGGCTTT |
| TGFB1-R | CGGTAGTGAACCCGTTGATG |
| PVR-F | CCGTCCAGGTCAAAGGTACAG |
| PVR-R | GGGGTCTTCATCCATTGGGG |
| LGALS9-F | GGACGGACTTCAGATCACTGT |
| LGALS9-R | CCATCTTCAAACCGAGGGTTG |
| CD274-R | GCCGACTACAAGCGAATTAC |
| CD274-R | TCTCAGTGTGCTGGTCACAT |

**
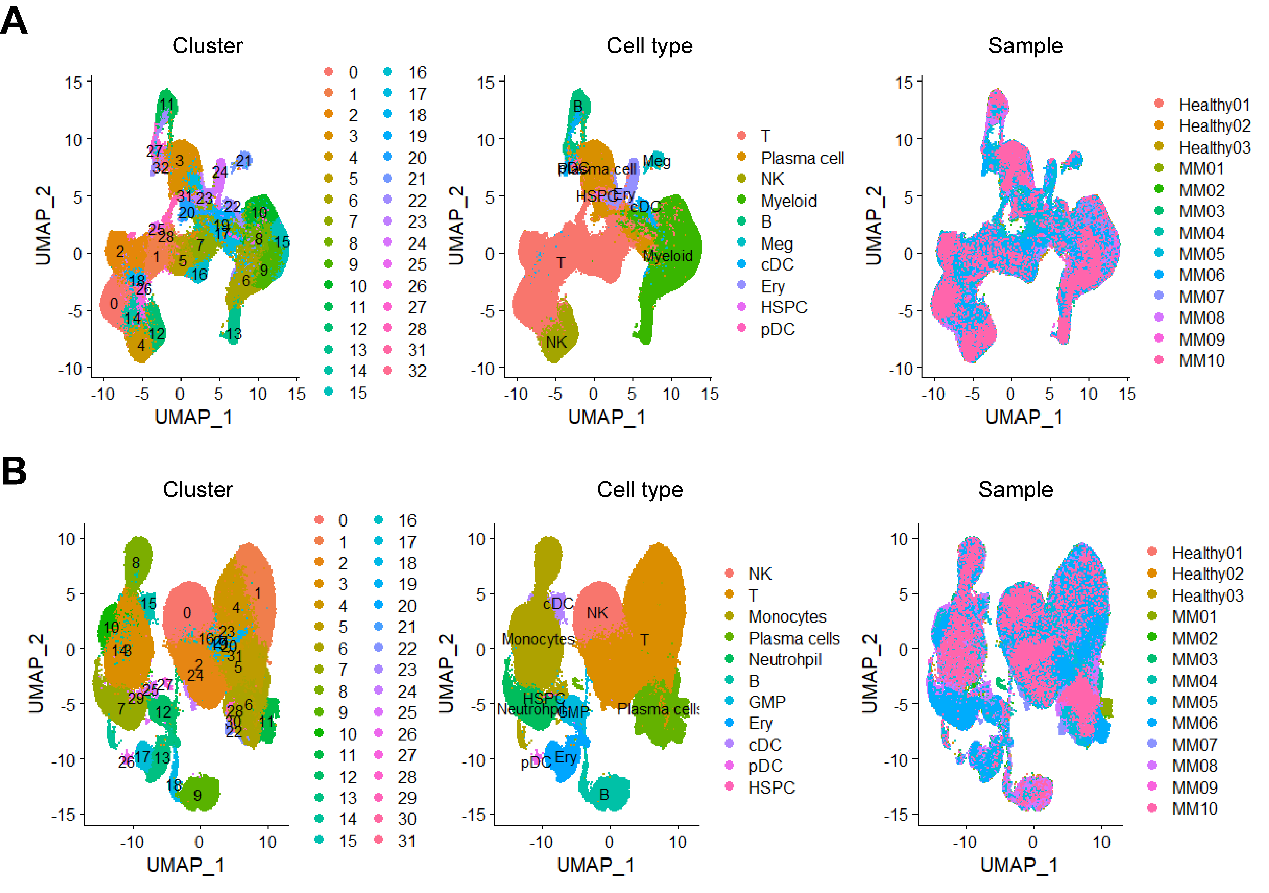
**

**Fig. S1 Comparison of integration performance by different methods.** **A** UMAP embeddings of the integration results performed by Seurat Integration [3], and cells were colored by cluster (left), cell type (middle) and sample (right). **B** UMAP embeddings of the integration results performed by Harmony [4], and cells were colored by cluster (left), cell type (middle) and sample (right).

**
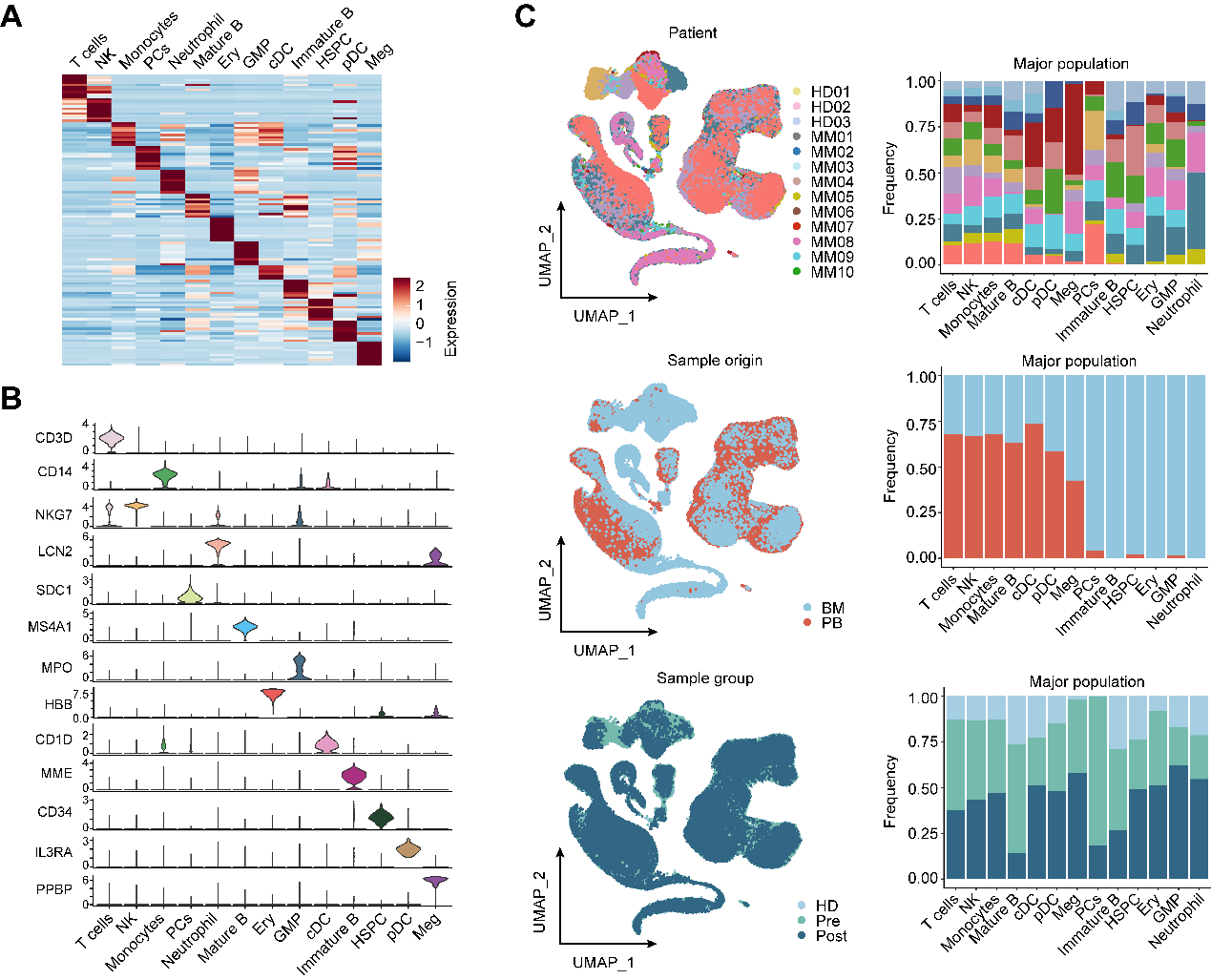
**

**Fig. S2 Single cell assessment of MM and healthy donors.** **A-B** Heat map (**A**) and Violin plots (**B**) showing canonical gene expression markers to define major cell types in healthy donors (HD) and MM. **C** UMAP plot of single cells profiled in the presenting work after fastMNN integration colored by patients (left) and frequency of each immune cell type in patients (right). **D** UMAP plot of single cells colored by sample origin (left) and frequency of each immune cell type in BM and PB (right). **E** UMAP plot of single cells colored by sample group (left) and frequency of each immune cell type in different groups (right)

**
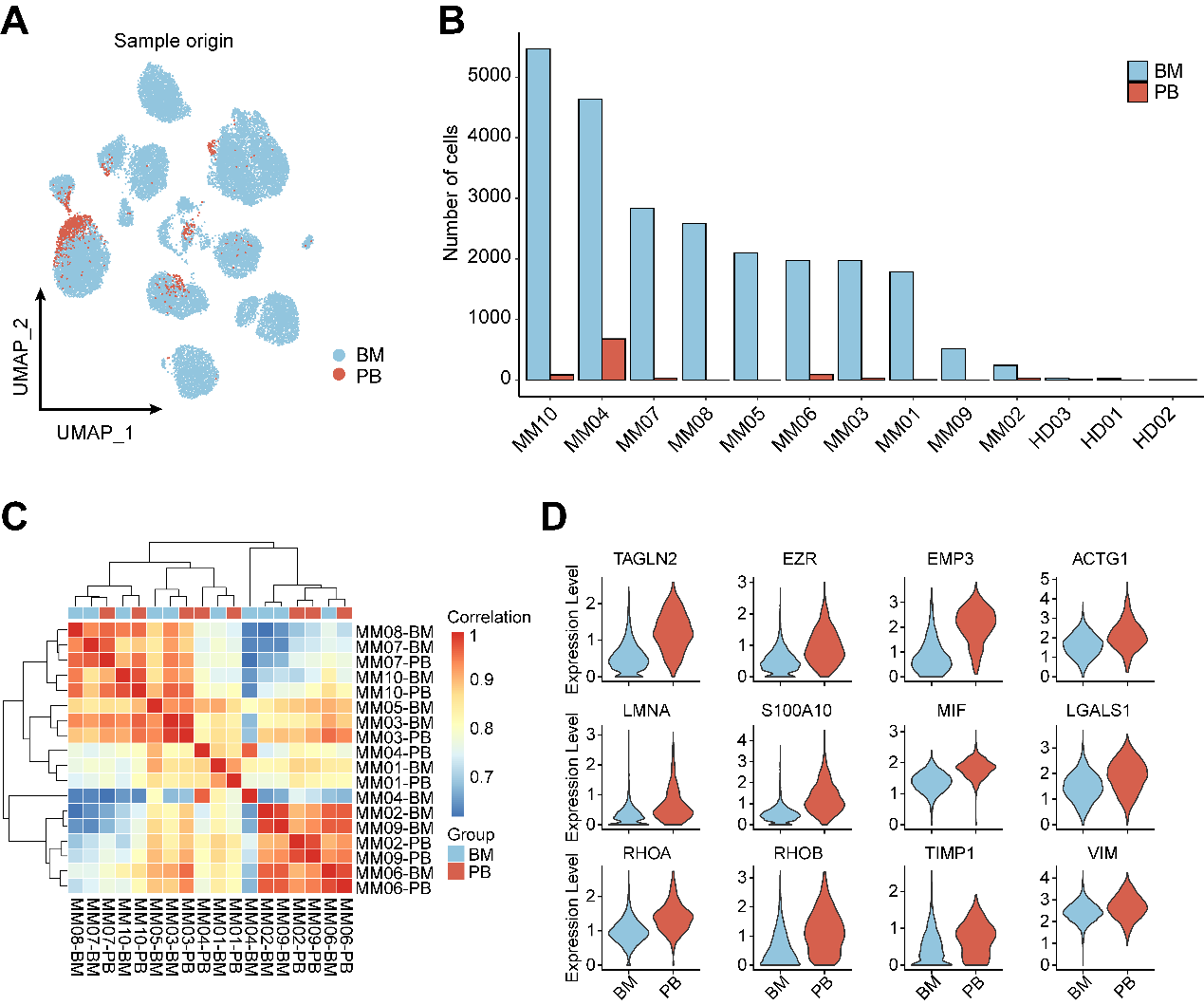
**

**Fig. S3 Characterization of PCs from BM and PB. A** UMAP embedding of PCs colored by sample origin (BM and PB). **B** PCs number in BM and PB from each MM patient and healthy donor. **C** Hierarchical clustering of correlation between BM and PB per patient. **D** Violin plots of gene expression levels in BM and PB from MM04 patient.

**
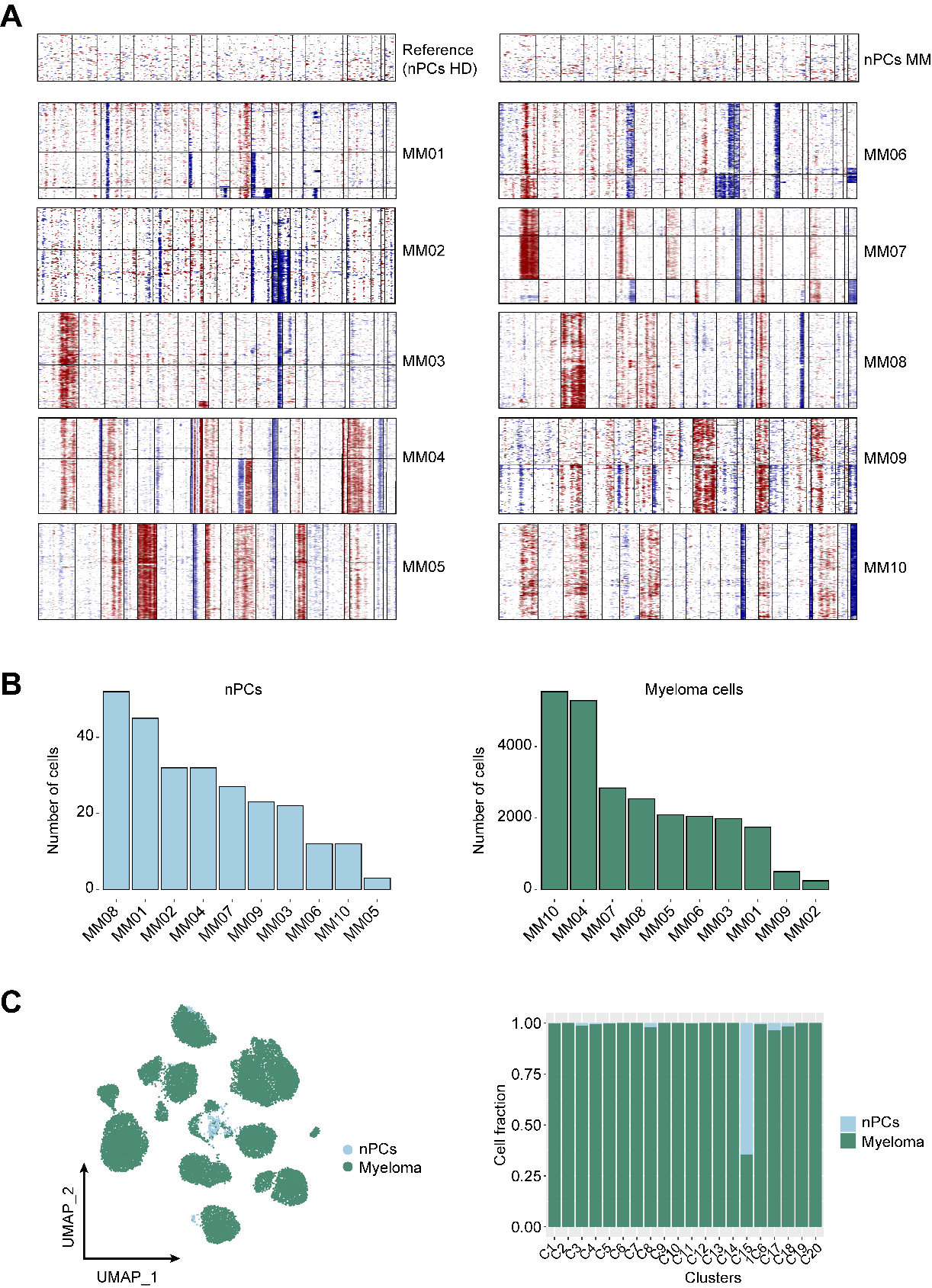
**

**Fig. S4 Copy number alterations (CNAs) analysis from scRNA-seq data. A** Heat map of CNAs signal per patient normalized against nPCs derived from the healthy donors as reference using the InferCNV package. Top left, nPC reference from healthy donors; top right, nPCs from MM samples. **B** Bar plot of nPC (left) and myeloma cells (right) numbers per MM patient. **C** UMAP embedding (left) and cell faction in each cluster (right) of nPCs and myeloma cells derived from MM patients and healthy donors. **D** Heat map of average gene expression of PCs from healthy donors and MM patients with different cytogenetics. **E** Expression of multiple established myeloma gene expression classifiers in MM patients with different cytogenetics.

**
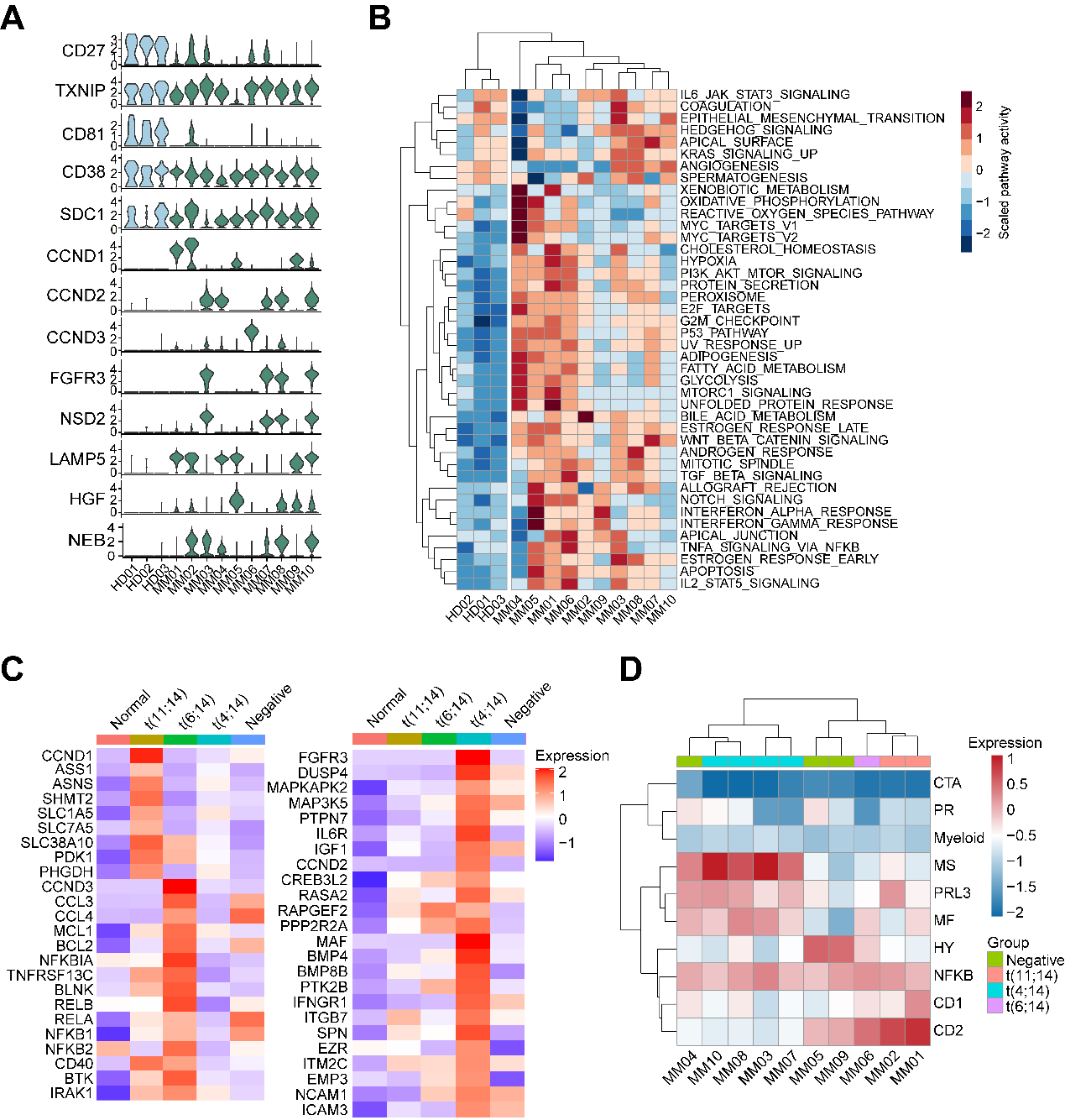
**

**Fig. S****5 Characterization of nPCs from healthy donors and myeloma cells from MM. A** Violin plots showing normalized expression of canonical plasma cell genes and common MM driver genes in 3 healthy donors (HD), and 10 MM individuals. **B** Heat map showing difference in HALLMARK pathway activities scored by GSVA between HD and MM.

**
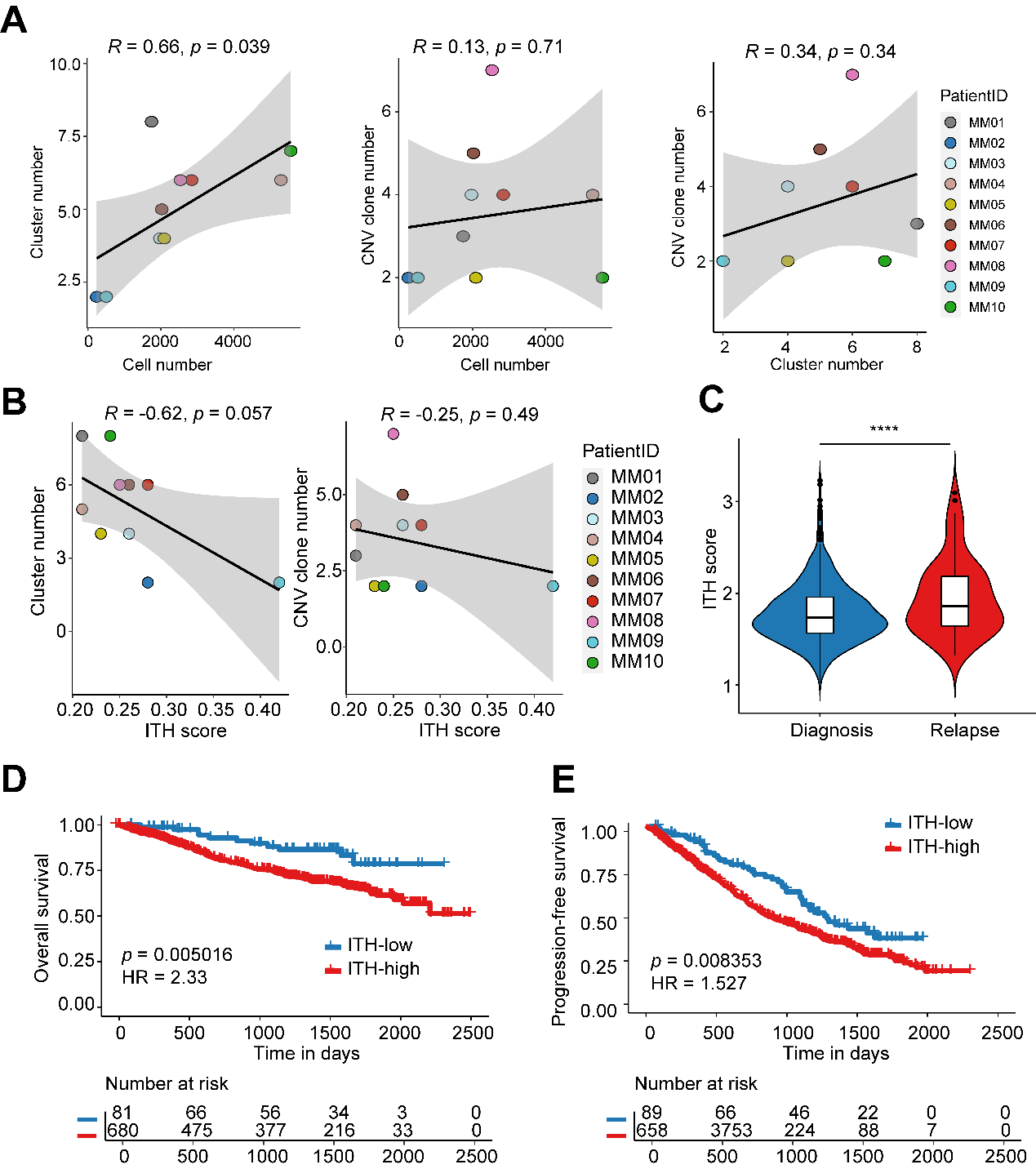
**

**Fig. S6 Intra-tumor heterogeneity of MM. A** Scatterplots showing relationships between number of cells, clusters and CNA clones per patient. Regression line and 95% confidence interval are indicated. The Pearson’s correlation coefficient was *R* = 0.61 (*p* = 0.039, clusters vs. cells), *R* = 0.13 (*p* = 0.71, clones vs. clusters) and *R* = 0.34 (*p* = 0.34, clones vs. cells). **B** Scatterplots showing relationships between ITH score, clusters (left) and CNA clones (right) per patient. Regression line and 95% confidence interval were indicated. The Pearson’s correlation coefficient was *R* = -0.62 (*p* = 0.057, ITH score vs. clusters), *R* = -0.25 (*p* = 0.49, ITH score vs. clones). **C** Violin plot of ITH score in diagnosis and relapse patients from CoMMpass database (Diagnosis, n=764; Relapse, n=80). (Wilcoxon test, *****p*<0.0001) **D** KM plot and analysis for OS comparing NDMM patients in CoMMpass data with high ITH score (red) and patients with low ITH score (blue) (logrank test, two-sided *p* = 0.005016, HR = 2.33). **E** KM plot and analysis for PFS comparing NDMM patients in CoMMpass data with high ITH score (red) and patients with low ITH score (blue) (logrank test, two-sided *p* = 0.008353, HR = 1.527).

**
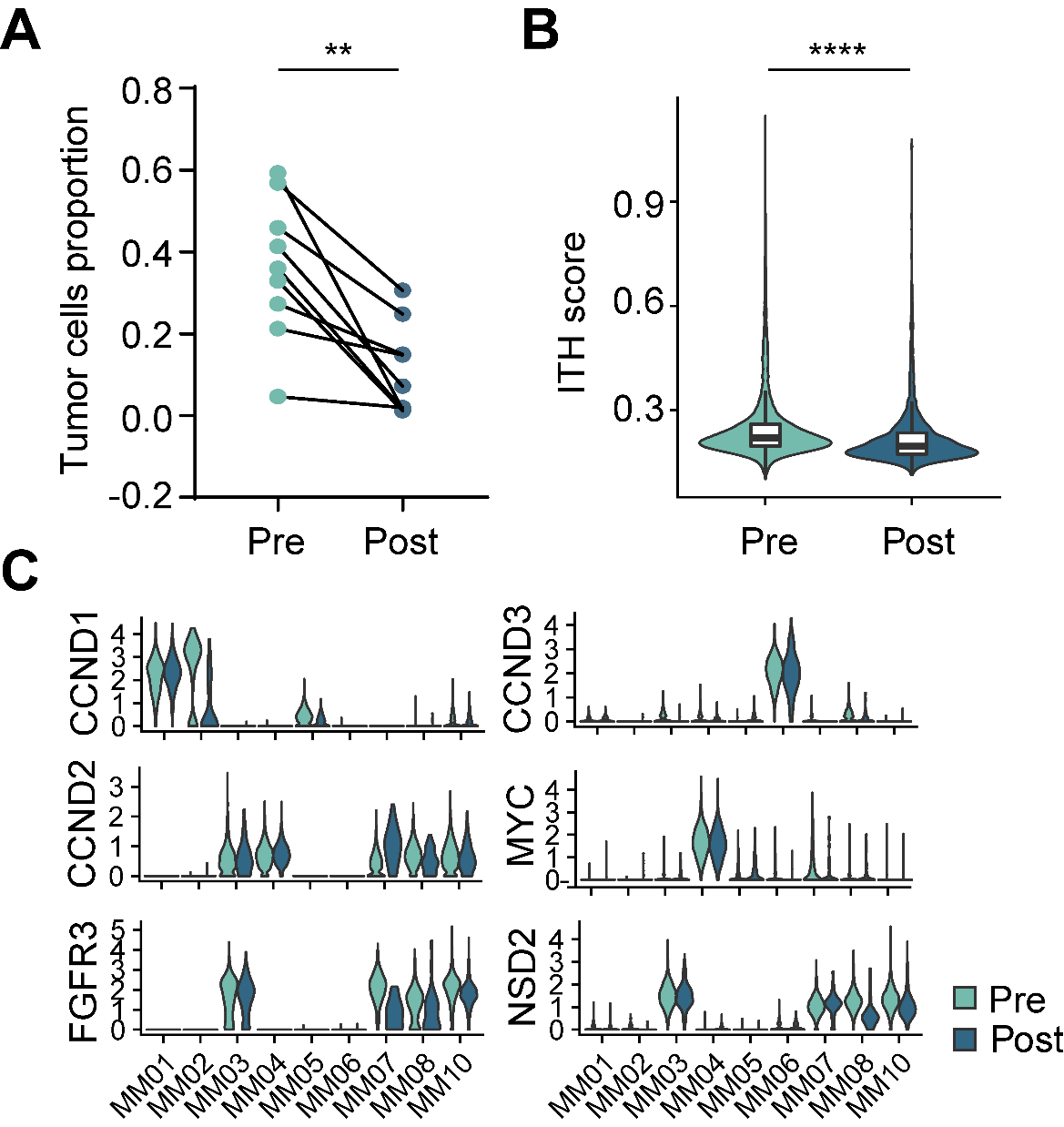
**

**Fig. S7 Analysis of myeloma cells pre/post- treatment.** **A** Dot plot illustrating the fraction of tumor cells, in 9 matched pre and post samples. **B** Violin plot showing ITH score in pre/post-treatment samples. **C** Violin plots of gene expression levels of driver genes pre/post-treatment in each MM patient. Statistical analysis in **A, B** were performed by Wilcoxon test, ***p*<0.01, *****p*<0.0001.

**
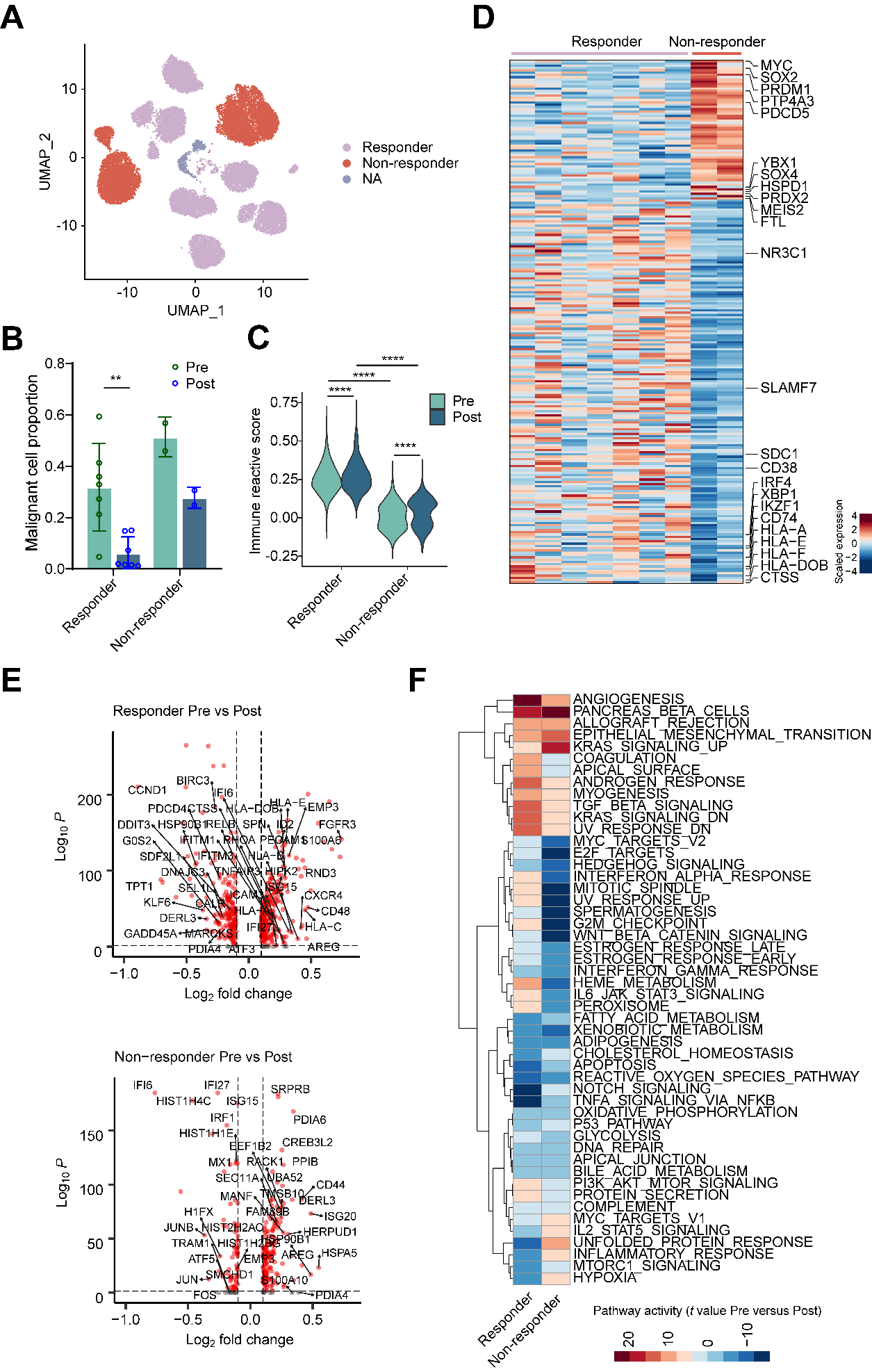
**

**Fig. S8 Analysis of myeloma cells in responders and non-responders.** **A** UMAP embedding of myeloma cells colored by treatment response (Responder, n = 7, Non-responder, n = 2, NA = 1). **B** Bar plot showing the proportion of malignant cells in responders and non-responders pre- and post-treatment.

**C** Violin plot of immune reactive score in responders and non-responders pre and post treatment. **D** Heat map illustrating average expression of representative DEGs in myeloma cells from pre-treatment samples of responders and non-responders. **E** Volcano plots of differentially expressed genes using two-sided Wilcoxon rank-sum test in myeloma cells from responders (up) and non-responders (bottom) (Pre vs Post). Thresholds for differential expression were *p*-value < 0.05 (Bonferroni-adjusted) and logFC > 0.1. **F** Differences in pathway activities scored per cell using GSVA, in myeloma cells derived from pre- and post-treatment samples. Shown are *t* values from a linear model for difference between pre-treated cells from post-treated cells. Statistical analysis in **B** and **C** were performed by Wilcoxon test, ***p*<0.01, *****p*<0.0001.

**
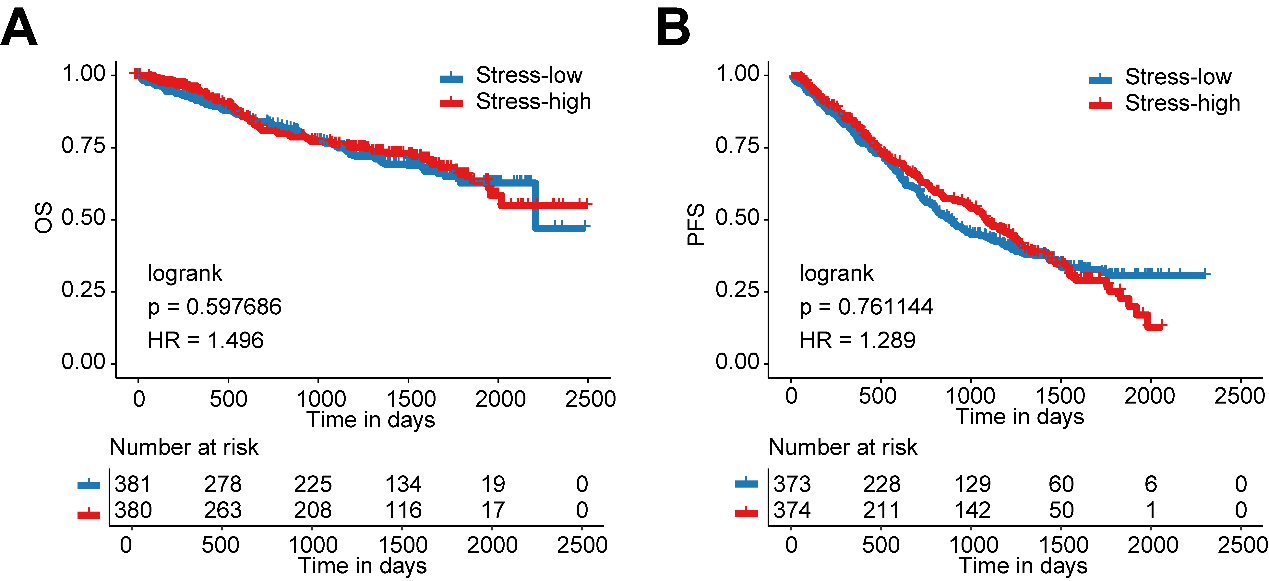
**

**Fig. S9 Impacts of stress program on prognosis of MM patients. A** KM plot and analysis for OS comparing NDMM patients in CoMMpass data with high stress score (red) and patients with low stress score (blue). **B** KM plot and analysis for PFS comparing NDMM patients in CoMMpass data with high stress score (red) and patients with low stress score (blue).

**
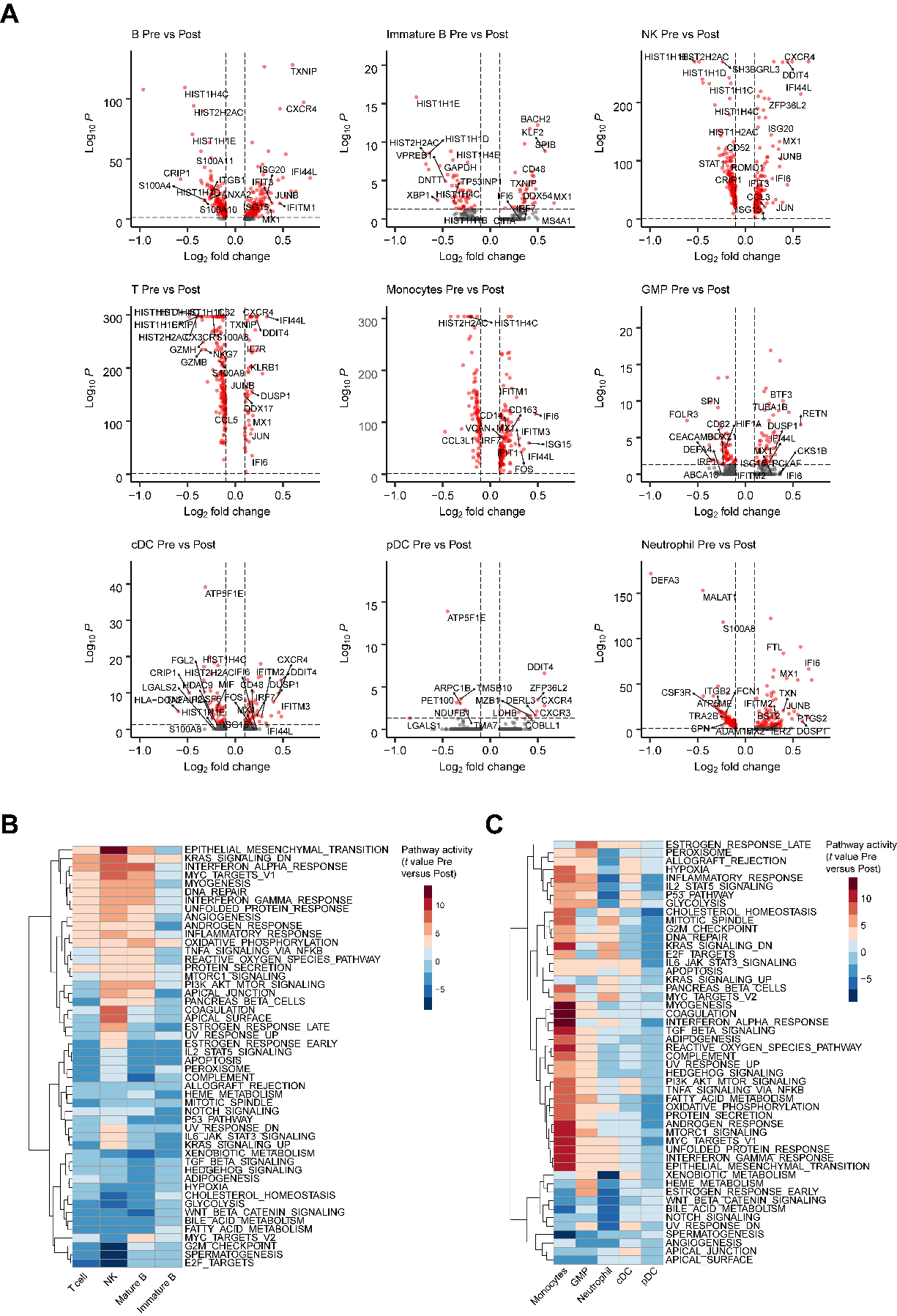
**

**Fig. S10 Analysis of** **immune cells pre/post-treatment.** **A** Volcano plots of differentially expressed genes using two-sided Wilcoxon rank-sum test in indicated immune cells from pre- and post-treatment samples. Thresholds for differential expression were *p*-value < 0.05 (Bonferroni-adjusted) and logFC > 0.1. **B-C** Differences in pathway activities scored per cell using GSVA, in lymphoid (**B**) and myeloid cell subsets (**C**) derived from pre- and post-treatment samples. Shown are *t* values from a linear model for difference between pre-treated cells from post-treated cells.

**
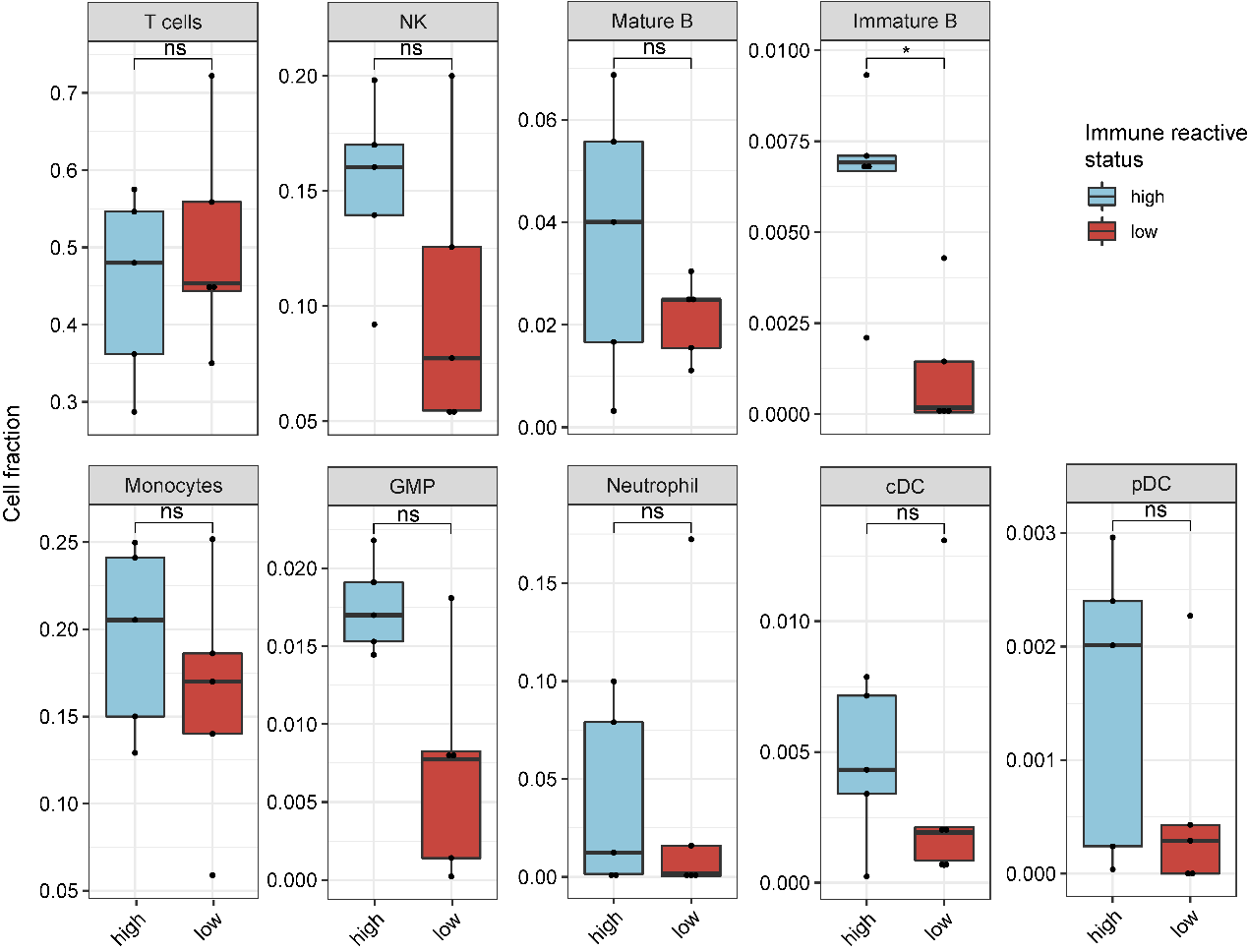
**

**Fig. S11 Immune cell fractions**. Box plot for the comparison of immune cell type fractions between immune reactive-high patients and low patients. Statistical analysis was performed by Wilcoxon test (ns not significant, **p*< 0.05).

**
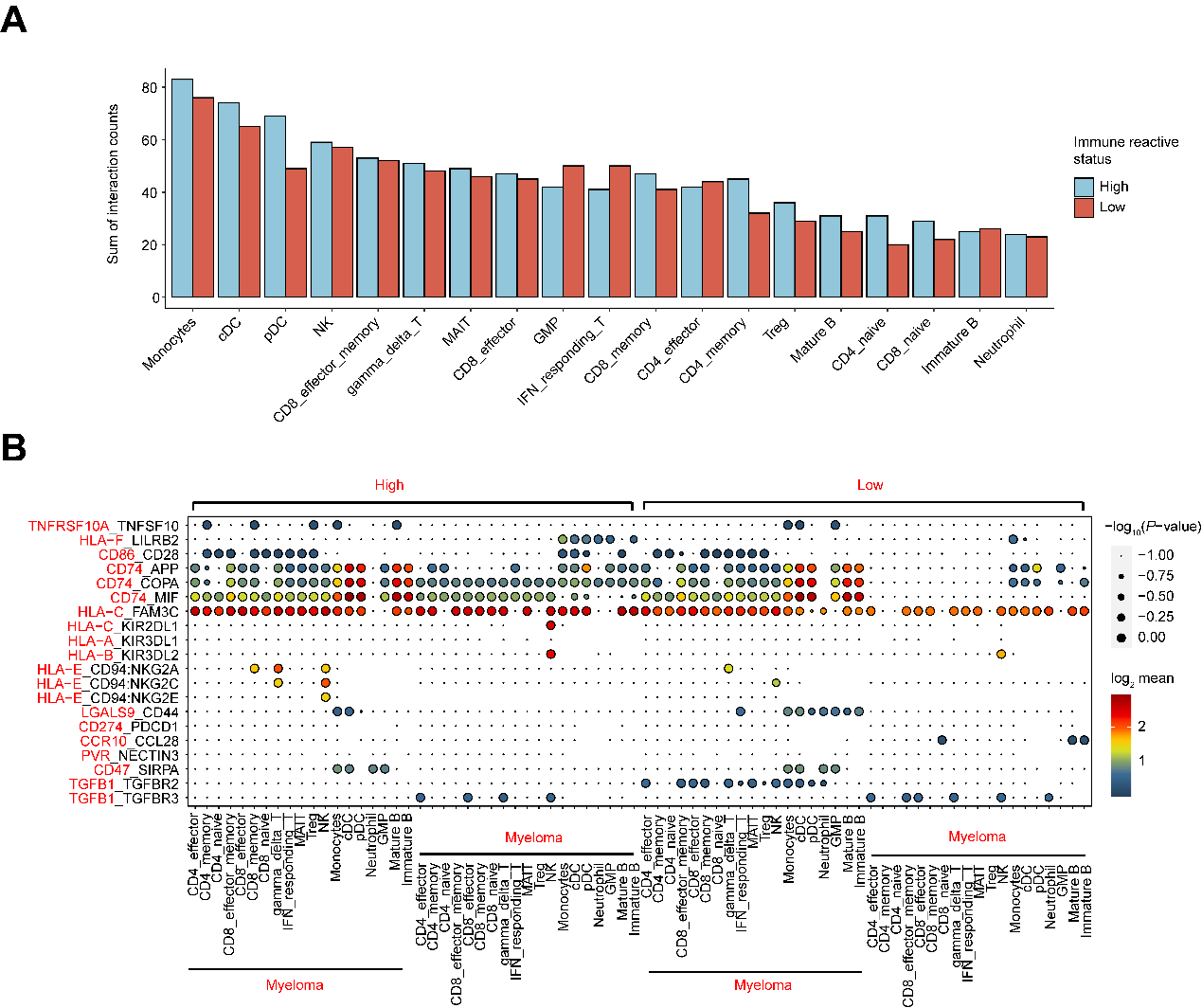
**

**Fig. S12 Cellular interactions in MM with different immune reactive status. A** Bar plot showing sum of interaction in BM samples of MM individuals with high or low immune reactive status. **B** Bubble plot showing the interactions between malignant cells and other cell types in MM patients with high or low immune reactive status, based on selected ligand and receptor pairs.

**
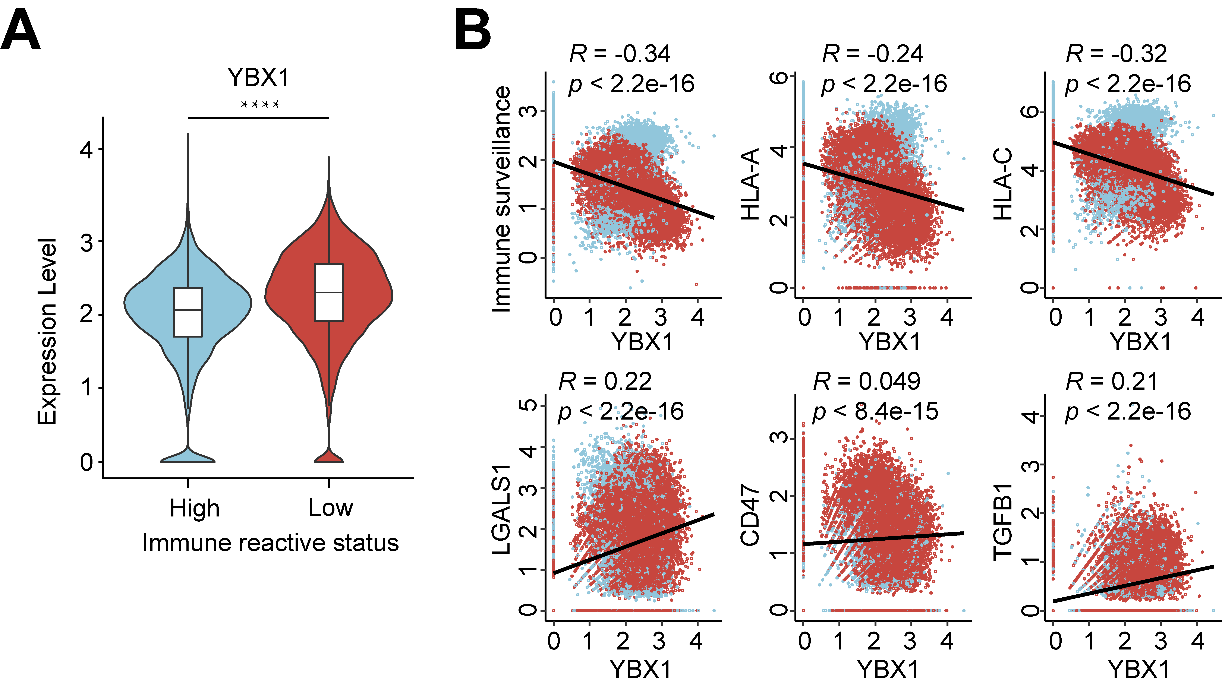
**

**Fig. S13 Correlations of YBX1 expression with immune response and escape in MM. A** Violin plot of YBX1 expression in MM patients with high or low immune reactive score. (Wilcoxon test, *****p*<0.0001). **B** Correlations of YBX1 expression with immune surveillance and escape.

**
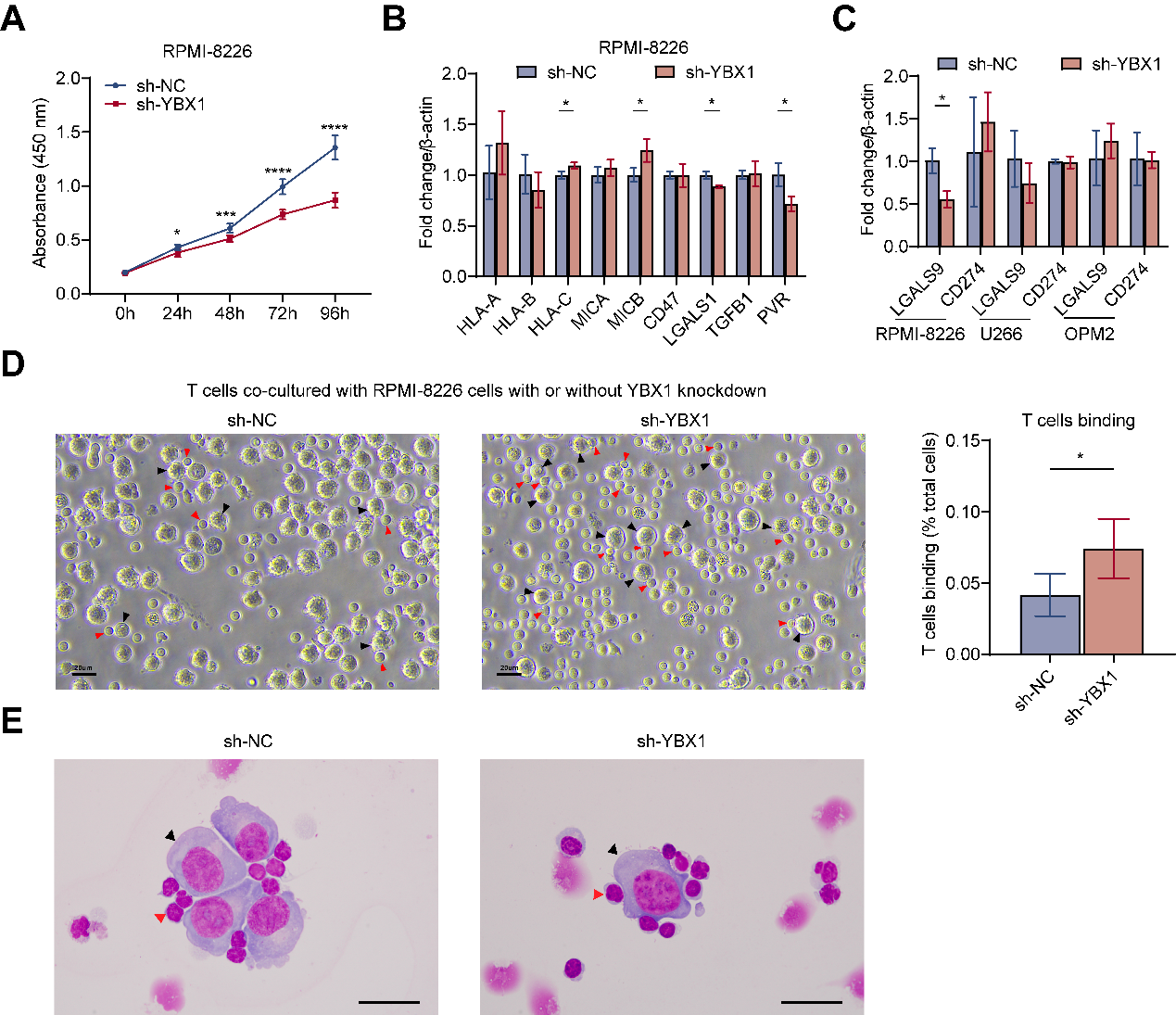
**

**Fig. S14 RPMI-8226 cells proliferation curve, target genes expression and co-culture with T cells. A** Proliferation curve of RPMI-8226 cell line with transduction of sh-YBX1 or sh-NC using CCK-8 assay. **B** qPCR analysis showing the mRNA expression of key targets in RPMI-8226 cell line after transduction of sh-YBX1 or sh-NC. **C** qPCR analysis showing the mRNA expression of LGALS9 and CD274 in U266, OPM2 and RPMI-8226 cell lines after transduction of sh-YBX1 or sh-NC. **D** Representative microphotograph images (left and middle) and quantification (right) for the percentage of T cells binding myeloma cells after 12 hours co-culture. **E** Wright-Giemsa staining showing the morphology of myeloma and T cells. Red and black arrowheads pointed to T cells and myeloma cells respectively. sh-NC n = 4, sh-YBX1 n=4. Scale bar = 20 μm. Error bars denoted mean ± SD. Two-tailed Student *t* test: **p*< 0.05, ****p*< 0.001, *****p*< 0.0001.

**
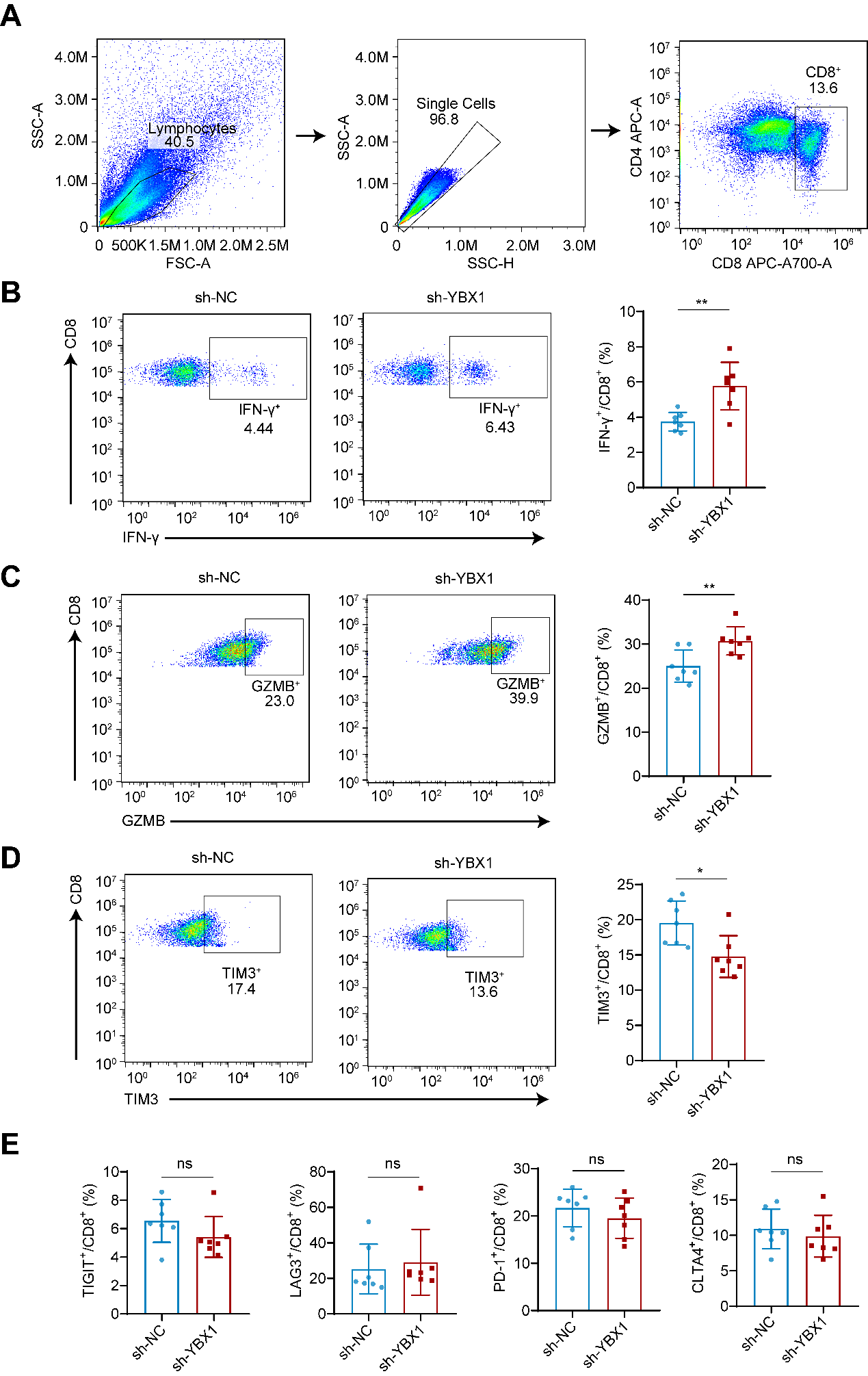
**

**Fig. S15** **Flow cytometric analysis of CD8^+^ T cells function co-cultured with RPMI-8226 cells.** CD3^+^ T cells from healthy donors (⁠n = 7) were isolated and co-cultured with control RPMI-8226 cells (sh-NC) or RPMI-8226 cells with YBX1 knockdown (sh-YBX1). After 5 days of co-culture, flow cytometry was performed to analyze expression of exhausted and cytotoxic markers in CD8^+^ T cells. **A** FACS gating strategy for CD8^+^ T cells from the co-culture suspension. **B-D** Representative flow cytometry plots (left) and quantitative frequencies (right) of IFN-γ^+^CD8^+^ cells (**B**), GZMB^+^CD8^+^ cells (**C**), TIM3^+^CD8^+^ cells (**D**) co-cultured with RPMI-8226 cells with sh-NC or sh-YBX1. **E** Quantitative frequencies of TIGIT^+^CD8^+^ cells, LAG3^+^CD8^+^ cells, PD-1^+^CD8^+^ cells, CTLA4^+^CD8^+^ cells co-cultured with RPMI-8226 cells with sh-NC or sh-YBX1. Error bars denoted mean ± SD. Two-tailed Student *t* test: ns not significant, **p*< 0.05, ***p*< 0.01. FACS: fluorescence-activated cell sorting.

**References**

1. Liberzon A, Birger C, Thorvaldsdottir H, Ghandi M, Mesirov JP, Tamayo P. The Molecular Signatures Database (MSigDB) hallmark gene set collection. Cell Syst. 2015;1(6):417-425.

2. Sun Y, Wu L, Zhong Y, Zhou K, Hou Y, Wang Z, et al. Single-cell landscape of the ecosystem in early-relapse hepatocellular carcinoma. Cell. 2021;184(2):404-421 e416.

3. Stuart T, Butler A, Hoffman P, Hafemeister C, Papalexi E, Mauck WM, 3rd, et al. Comprehensive Integration of Single-Cell Data. Cell. 2019;177(7):1888-1902 e1821.

4. Korsunsky I, Millard N, Fan J, Slowikowski K, Zhang F, Wei K, et al. Fast, sensitive and accurate integration of single-cell data with Harmony. Nat Methods. 2019;16(12):1289-1296.
